# Network Analyses of Maternal Pre- and Post-Partum Symptoms of Depression and Anxiety

**DOI:** 10.3389/fpsyt.2020.00785

**Published:** 2020-08-06

**Authors:** Desiree Y. Phua, Helen Chen, Yap Seng Chong, Peter D. Gluckman, Birit F. P. Broekman, Michael J. Meaney

**Affiliations:** ^1^Singapore Institute for Clinical Sciences, Agency for Science, Technology and Research (ASTAR), Singapore, Singapore; ^2^Department of Psychological Medicine, KK Women’s and Children’s Hospital, Singapore, Singapore; ^3^Yong Loo Lin School of Medicine, National University of Singapore, Singapore, Singapore; ^4^Centre for Human Evolution, Adaptation and Disease, Liggins Institute, University of Auckland, Auckland, New Zealand; ^5^Amsterdam UMC and OLVG, VU University, Amsterdam, Netherlands; ^6^Sackler Program for Epigenetics & Psychobiology, McGill University, Montreal, QC, Canada; ^7^Department of Psychiatry, Douglas Mental Health University Institute, McGill University, Montreal, QC, Canada

**Keywords:** maternal psychopathology, positive mental health, bridging symptoms, perinatal, child development

## Abstract

**Background:**

Maternal mental health problems often develop prenatally and predict post-partum mental health. However, the circumstances before and following childbirth differ considerably. We currently lack an understanding of dynamic variation in the profiles of depressive and anxiety symptoms over the perinatal period.

**Methods:**

Depressive and anxiety symptoms were self-reported by 980 women at 26-week pregnancy and 3 months post-partum. We used network analysis of depressive and anxiety symptoms to investigate if the symptoms network changed during and after pregnancy. The pre- and post-partum depressive-anxiety symptom networks were assessed for changes in structure, unique symptom-symptom interactions, central and bridging symptoms. We also assessed if central symptoms had stronger predictive effect on offspring’s developmental outcomes outcomes at birth and 24, 54, and 72 months old than non-central symptoms. Bridging symptoms between negative and positive mental health were also assessed.

**Results:**

Though the depressive-anxiety network structures were stable during and after pregnancy, the post-partum network was more strongly connected. The central depressive-anxiety symptoms were also different between prenatal and post-partum networks. During pregnancy, central symptoms were mostly related to feeling worthless or useless; after pregnancy, central symptoms were mostly related to feeling overwhelmed or being punished. Central symptoms during pregnancy were associated with poorer developmental outcomes for the child. Anxiety symptoms were strongest bridging symptoms during and after pregnancy. The interactions between negative and positive mental health symptoms were also different during and after pregnancy.

**Conclusions:**

The differences between pre- and post-partum networks suggest that the presentation of maternal mental health problems varies over the peripartum period. This variation is not captured by traditional symptom scale scores. The bridging symptoms also suggest that anxiety symptoms may precede the development of maternal depression. Interventions and public health policies should thus be tailored to specific pre- and post-partum symptom profiles.

## Introduction

Maternal depression and anxiety associate with psychosocial impairments for the mother and predict multiple developmental outcomes in the offspring [e.g., (1–4)]. The World Health Organization (5) reported about 10% of women in developed countries, and 1/3 of women in developing countries suffer from severe perinatal mental health issues. Moreover, clinical and sub-clinical levels of maternal symptoms of depression and anxiety influence the neurodevelopment of the offspring and increase the risk of future psychopathology for the child (1, 6, 7). The economic cost of poor prenatal maternal mental health is estimated at £8.1 billion of which 72% is associated with child outcomes (8).

Longitudinal trajectory analyses clearly show that the measures of maternal distress that predict the increased risk for depression in the offspring are highly stable over the perinatal period (9–11). These large cohort analyses reveal that only a small percentage of mothers show a major change in symptom levels following parturition. Recent systematic reviews of depressive (12) and anxiety symptoms (13) over the peripartum period also reveal that the most commonly reported profiles are those of stable low, medium, or high symptom levels. This stability is somewhat surprising, as the demands of motherhood vary considerably prior to and following childbirth. The post-partum period is a transition to motherhood with new roles, responsibilities, and adjustments (14). Even for multiparous mothers, there is a transition to having an additional child for whom to care (15).

Changes in the social context of motherhood may influence the presentation of depressive and anxiety symptoms (16). Moreover, the factors that predict poor prenatal mental health differ from those of the early post-partum period (17). The demographic variables associated with depression during pregnancy and post-partum differ (18). However, studies on pre- and post-partum mental health often report total scores on self-report [e.g., (15)] or clinician-report symptom questionnaires [e.g., (19)] and do not inform on more qualitative differences in mental health symptoms. A longitudinal study on the different anxiety disorders showed that the presentation of anxiety symptoms changed during the peripartum phase (19). There is a need to extend analyses beyond scale scores to provide a deeper analysis of the depressive and anxiety symptoms in the pre- and post-partum phases of motherhood to advance interventions tailored to the maternal demands of a dynamic peripartum period.

An examination of individual depressive-anxiety symptoms allow us to have a deeper understanding of maternal mental health across the peripartum period. This study aims to examine the change in structure of peripartum mental health from the network perspective. Using the network approach, we can go beyond merely looking at how the severity of depressive-anxiety symptoms change (20) as mothers transit from pregnancy to motherhood. In this study, we sought to study the reinforcing loops among symptoms, thus giving us insights into how the mechanisms of maternal mental health may change with the external circumstances (21).

### Network Analysis

Network analysis is increasingly used to study psychopathology [see (22) for review], as it allows for an appreciation of mental health as an emergent property of the interaction of multiple symptoms (23, 24). This approach contrasts with the traditional latent variable analyses that presume an unobserved underlying psychiatric disorder that affects the presentation of symptoms. In network analysis, the disorder is the combination of symptoms and interactions among these symptoms (24). Symptoms that are strongly connected to each other form strong networks, resulting in a persistent activation of the network (or disorder) even after the removal of the initial activation stressor (25). Moreover, due to the strong interactions among symptoms, mild stressors are likely to propel the individual into a clinical state of psychopathology (26).

This symptom-oriented approach extends beyond describing mental health as sum scores of symptoms to provide deeper characterization of symptom-symptom interactions that is fundamental to establishing an evidence-based dimensional approach to mental health. Effective therapies often concentrate on a selected number of symptoms, which lead to improvement in other “untargeted” symptoms and an overall improvement of mental health (27). This interaction among symptoms is not aligned with the common cause perspective that is assumed in computation of sum scores (28). Clinically, an understanding of such symptom-symptom interactions *via* network analyses allows interventions to be tailored for maximum effectiveness (29).

Depression is a heterogeneous disorder with respect to etiology (30, 31), symptom profiles (32), and response to treatment (33). Maternal depression is similarly heterogeneous in latent profiles of symptoms (34) and trajectories of symptoms scores [e.g., (35–37)]. An examination of maternal depression as symptoms allows for a deeper understanding of the heterogeneity in how the symptoms affect each other. Moreover, knowing how central symptoms change informs on the reasons underlying different trajectories and etiological profiles (38) of psychopathology across the peripartum period, thus informing intervention programs.

Depression is often comorbid with anxiety symptoms [e.g., (39–41)]. This comorbidity extends to women during and after pregnancy (42). From the network perspective, comorbid conditions arise because of *bridging symptoms* that are strongly associated with symptoms of both conditions (27, 43). The identification of bridging symptoms in maternal mental health allows us to understand why some women exhibit both depressive and anxiety symptoms. This in turn facilitates the development of effective interventions for mothers with comorbid conditions (42). This is essential since comorbid depressive and anxiety symptoms associate with the worst neonatal outcomes (44) with greater treatment resistance, severity of symptoms, and persistence of poor mental health (42).

### Present Study

The present study compared maternal depression and anxiety symptom networks during the prenatal (i.e., 26 weeks gestation) and postnatal (i.e., 3 months post-partum) periods. We were interested in changes in the (i) network structures, (ii) symptom-symptom interactions (i.e., edge weights), (iii) central symptoms as indicated by the expected influence (EI) indices, and (iv) bridge symptoms that connected depression and anxiety. We also tested the validity of central symptoms as predictors of poor child outcomes. Poor maternal mental health during pregnancy has strong implications for offspring development. However, if some symptoms are more central to maternal psychopathology, do these symptoms have greater impact on fetal and child development?

We were also interested in how specific positive mental health symptoms may be associated with depressive and anxiety symptoms. Negative and positive mental health are not merely opposite ends of a single continuum. They are independent constructs (45, 46) with distinct consequences on parenting and child development [see (47) for review]. Past studies also found enhanced positive mental health when women transit to motherhood (48, 49). As such, positive mental health symptoms were subsequently added to the depressive-anxiety networks for a more comprehensive understanding of maternal mental health (48) in the current study. This approach allowed for a holistic perspective on how relationships between negative and positive mental health changed over time (50). We examined which positive mental health symptoms were significant bridges (if any) to the depressive or anxiety symptoms and whether these bridging symptoms changed after pregnancy.

## Materials and Methods

### Participants

Pregnant women were recruited at ~11-week pregnancy (*N* = 1150) at two major public maternity clinics for a longitudinal birth cohort study [Growing Up in Singapore Towards Healthy Outcomes, GUSTO; (51)]. Participants who conceived through *in vitro* fertilization, had chronic health conditions, or were pregnant with twins were excluded. Participants who gave birth prematurely (<36 weeks) were also excluded. Mental health was assessed at 26 weeks of pregnancy during routine obstetric visits (*n* = 1119) and at 3 months post-partum (*n* = 774). In the current sample, we also excluded participants who did not answer the English version of the questionnaires (*n* = 139). Only complete cases of both the pre- (*n* = 981) and post-partum (*n* = 652) measurements were included in the network analyses (final *n* = 491; see **Table S1** for demographic data). Participants were invited to the laboratory with their child when the child was 24, 54, and 72 months old.

### Measurements

#### Depressive and Anxiety Symptoms

Depressive symptoms were assessed with the Beck Depression Inventory [BDI; (52)] and the Edinburgh Postnatal Depression Scale [EPDS; (53)]. The BDI consists of 21 sets of descriptions of depressive symptoms. Each set has four to seven statements describing the severity of a depressive symptom over the past 2 weeks. Participants selected the statement that best described how they felt over the past 2 weeks. The EPDS consists of 10 items measuring depressive symptoms. Participants rated the extent each EPDS item described their feeling for the past week on a four-point Likert scale. The use of two common depression scales allow for more comprehensive coverage of perinatal depressive symptoms as the scales measure different aspects of depression (54).

Anxiety and positive mental health symptoms were assessed using the Trait Anxiety subscale of the State-Trait Anxiety Inventory [STAI; (55)]. STAI has been validated to be a reliable measurement of maternal anxiety during pregnancy (56). Though the positively worded items were commonly reversed-coded and included as part of the measure of anxiety, studies have shown that these items are not merely absence of anxiety; they are indicators of a positive mental health that is independent from anxiety (57–60). Preliminary exploratory graph analysis of the STAI items replicated the two factors model in both prenatal and post-partum measurements (**Figure S1** in Supplementary Material). The two factors separated the negatively and positively worded items to different latent clusters. Participants rated on a four-point Likert scale on how much each item described how they felt in general.

The endorsement rates of the individual BDI, EPDS, and STAI items are shown in **Table S2**.

#### Birth Outcomes

We used gestational age (*M* = 38.43 weeks, *SD* = 1.33 weeks), birth weight (*M* = 3119.27 grams, *SD* = 449.09 grams), and birth length (*M* = 48.86 cm, *SD* = 2.31 cm) as indicators of birth outcomes. Data on gestational age, birth weight and birth length were retrieved from the hospital delivery records. Gestational age was determined from the first trimester dating ultrasound scan. The birth weight and birth length were measured and recorded within 72 hours of birth. These measures were used to examine the predictive value of central prenatal depressive-anxiety symptoms.

#### Child Outcomes

Mothers rated their child on the Child Behavioral Checklist [CBCL; (61)] during the 24-month (2 years old) visit. When the child was 54 months old (4.5 years), the child’s overall cognitive ability was assessed on the Kaufman Brief Intelligence Test [KBIT; second edition; (62)]. At 72 months old (6 years), the child completed Spatial Working Memory (SWM) and Stop-Signal Task (SST). See **Supplementary Material** for more details.

### Statistical Analyses

#### Symptom Networks

The prenatal and post-partum depressive-anxiety symptom networks were jointly estimated using the Fused Graphical Lasso (FGL) in the *EstimateGroupNetwork* R package (63). The FGL estimates the two networks simultaneously to exploit similarities between the networks (64, 65). This joint estimation of the networks provides a more accurate estimation if the networks are indeed similar. If the two networks are very different, then the joint estimation will produce networks similar to independently estimated networks (66, 67).

The networks were visualized using the R-package *qgraph* (68). All edges between the symptoms (i.e., symptom-symptom interactions) represent partial correlation after controlling for the other symptoms in the network. The thickness of the edges represents the strength of the partial correlation (i.e., edge weight). The placement of the nodes was determined by the Fruchterman-Reingold algorithm, which placed nodes with the strongest connections in the center. For visual comparison purposes, the prenatal and post-partum network layouts were standardized by taking the average of the two networks’ layouts. The stability of the networks’ edges was tested with case-dropping bootstrap on 10,000 bootstrap samples and 150 sampling levels *via* the *bootnet* R package (69).

#### Networks Comparison

Changes in the general network structure, global network strength (i.e., overall absolute connectivity among the symptoms), and edge weights were tested statistically with 1,000 iterations *via* the *NetworkComparisonTest* package (70). As this was an exploratory study with no a-priori hypotheses and network comparison tests required high statistical power, we did not adjust for multiple testing to avoid type-II errors inflation (71). We also computed the Spearman correlation coefficient of the edge weights between the prenatal and post-partum networks; the coefficient is a measure of similarity between the two networks (66, 72). Lastly, we estimated a network of edge weights difference to visualize the edges that were different between the prenatal and post-partum networks. The edge weights in this difference-network represent changes in the partial correlations of the pairs of symptoms after pregnancy.

#### Symptom Centrality

EI index was used to identify core symptoms within the network. Core symptoms are symptoms that are highly connected to other symptoms and will have higher EI index. There are two types of EI: one-step (EI1) and two-step (EI2) indices. EI1 is the sum of the weights of the edges a node has with other nodes directly. It indicates how influential a node is in affecting its immediate neighbors. EI2 goes a step further by including the indirect influence a node can have *via* its neighbors (73). For example, node 1 is directly connected to nodes 2 and 3, but nodes 2 and 3 are not connected to each other. A change in node 2 may not affect node 3 directly, but due to its impact on node 1, node 3 will change in response to node 2. As such, EI2 assesses the cumulative influence a symptom has throughout the network (74). Both EI1 and EI2 indices were calculated with the *networktools* R package (75).

EI index is more appropriate for psychopathology networks due to the presence of positive and negative correlations (i.e., edges) within the networks. The traditional centrality measures, such as strength, calculates the sum of the absolute value of the edge weights without taking the sign into consideration (73). For example, a node with two connections of edge weights -10 and 10 will have a strength index of 20 but an EI1 index of 0. A change in the said node will not cause cascading change in the network because of the presence of positive and negative edges with equal weights. A negative EI1 index indicates that an increase in this symptom is associated with an overall decrease in the other symptoms. The EI indices will be highly similar to the strength index in networks with few negative edges (73).

To assess the validity of symptoms’ EI on the offspring’s development, we adopted the procedure from Elliot, Jones, and Schmidt (76). Zero-order spearman correlations between each symptom at prenatal measurement and each child outcome measure were calculated. These correlations were interpreted as the *predictive value* of the symptom. Simple linear regressions were then used to assess if symptoms with high EI also had high predictive value. In each regression, the independent variable was the prenatal standardized EI1, and the dependent variable was the predictive value. The beta coefficient indicates that the extent the EI1 of the symptom during pregnancy associates with the predictive utility of the symptom on offspring development.

#### Bridging Symptoms

Bridging symptoms are symptoms that connect two communities (e.g., depression and anxiety) within the network. Bridge EI indicates how much influence a node (e.g., a depressive symptom) has on the nodes in the other communities (e.g., anxiety symptoms). Bridge EI1 measures the direct influence a node has on the nodes of the other community. Bridge EI2 includes the secondary, indirect influence a node has *via* its immediate neighbors (74). The bridge centrality indices were calculated *via* the *networktools* R package (75). The stability of the EI1 and bridge EI1 indices were similarly tested with case-dropping bootstrap (69).

## Results

### Descriptive Statistics

**Table 1** shows the means, standard deviations, and paired t-tests of the BDI, EPDS, and STAI scale scores. There were significant differences between prenatal and post-partum depression scales (*p*s *<*.001). However, there were no significant differences in the STAI negative (i.e., anxiety) and positive scale scores (*p*s >.50) between the prenatal and post-partum time points.

**Table 1:** Mean, standard deviation (SD), cronbach alphas (*α*), and *t*-test results of mental health scale scores.

Scale	Prenatal			Post-partum			Paired *t*-test		
	Mean	SD	*α*	Mean	SD	*α*	*t*	*df*	*p*
BDI	8.46	6.04	.86	7.37	6.58	.89	3.73	490	<.001
EPDS	6.90	4.41	.84	6.14	4.49	.84	3.88	490	<.001
STAI-negative	17.76	4.72	.84	17.84	5.23	.87	-0.35	490	.71
STAI-positive	27.08	5.10	.91	27.20	5.49	.91	-0.52	490	.60

BDI, Beck Depression Inventory; EPDS, Edinburgh Postnatal Depression Scale; STAI, State-Trait Anxiety Inventory trait subscale. Negative refers to negatively worded items and is an indicator of anxiety level. Positive refers to positively worded items and is an indicator of positive mental health.

### Network Structures and Edges

**Figure 1:**
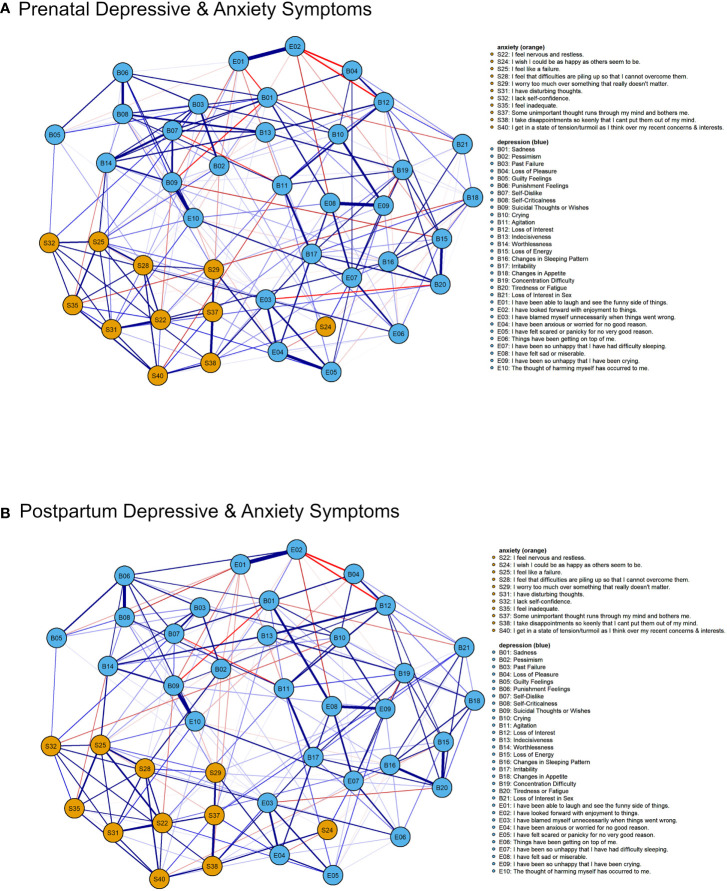
Prenatal **(A)** and post-partum **(B)** depressive-anxiety symptoms networks (jointly estimated). Weak edges with weights less than 0.05 are not shown. Blue edge represents positive association between the pair of symptoms (or nodes). Red edge represents negative association between the symptoms. The thickness of the edge represents the strength of association.

**Figure 1** shows the networks of prenatal (**Figure 1A**) and post-partum (**Figure 1B**) depressive and anxiety symptoms. A visual examination showed that the depressive-anxiety symptoms were densely connected in both prenatal and post-partum networks. There were no significant differences in the structure (*p* = .28) of the depressive-anxiety networks between prenatal and post-partum periods. However, the post-partum network was significantly more connected (global strength = 19.35) than the prenatal network (global strength = 18.55, *p* = .02).

**Figure 2:**
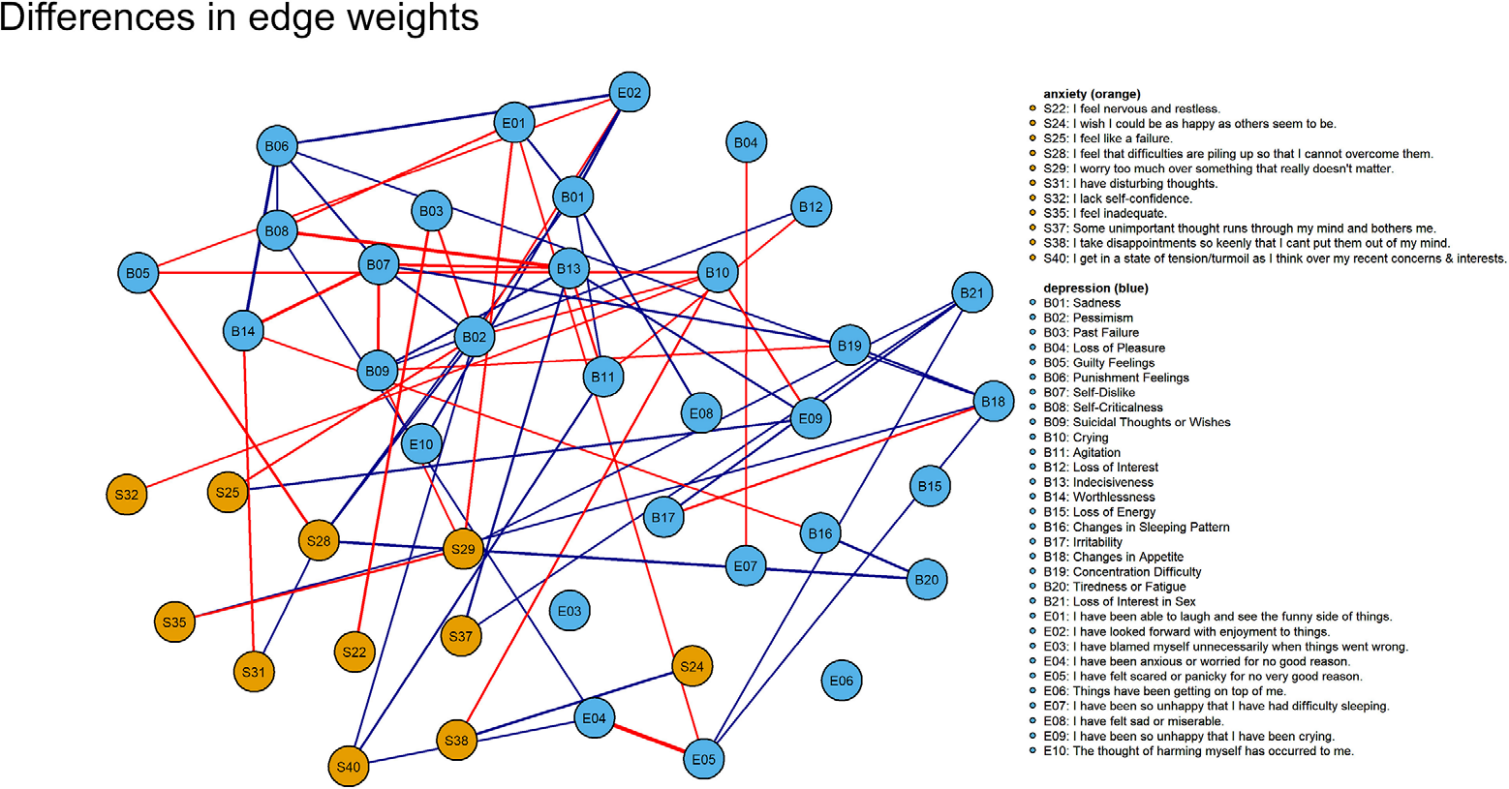
Network of differences in edge weights between the prenatal and post-partum depressive-anxiety networks. Blue edge denotes this edge was stronger in post-partum network than prenatal network. Red edge denotes this edge was stronger in the prenatal network. Weak differences with edge weight less than 0.05 are not shown here.

The edges were stable with correlation stability (CS) indices of 0.40 (prenatal network) and 0.42 (post-partum network), exceeding the recommended threshold of 0.25 (69). The edge weights in the prenatal and post-partum depressive-anxiety networks were significantly correlated (*r_s_* = .84, *p* <.001); the edges in the two networks were highly similar. Thirty-nine edges were statistically significant (*p*s < 0.05). Adapting the procedure from Fried et al. (66), we estimated a network based on differences of edge weights between the jointly estimated prenatal and post-partum depressive-anxiety networks (**Figure 2**). The strongest positive difference edge was *B14 (“Worthlessness”)-B06 (“Punishment feelings”)* with a difference of 0.11; this edge was stronger in the post-partum network. The strongest negative difference edge was *E5 (“Have felt scared or panicky for no very good reason”)- E4 (“Have been anxious or worried for no good reason”)* with a difference of -0.17.

### Symptoms Centrality

**Figure 3:**
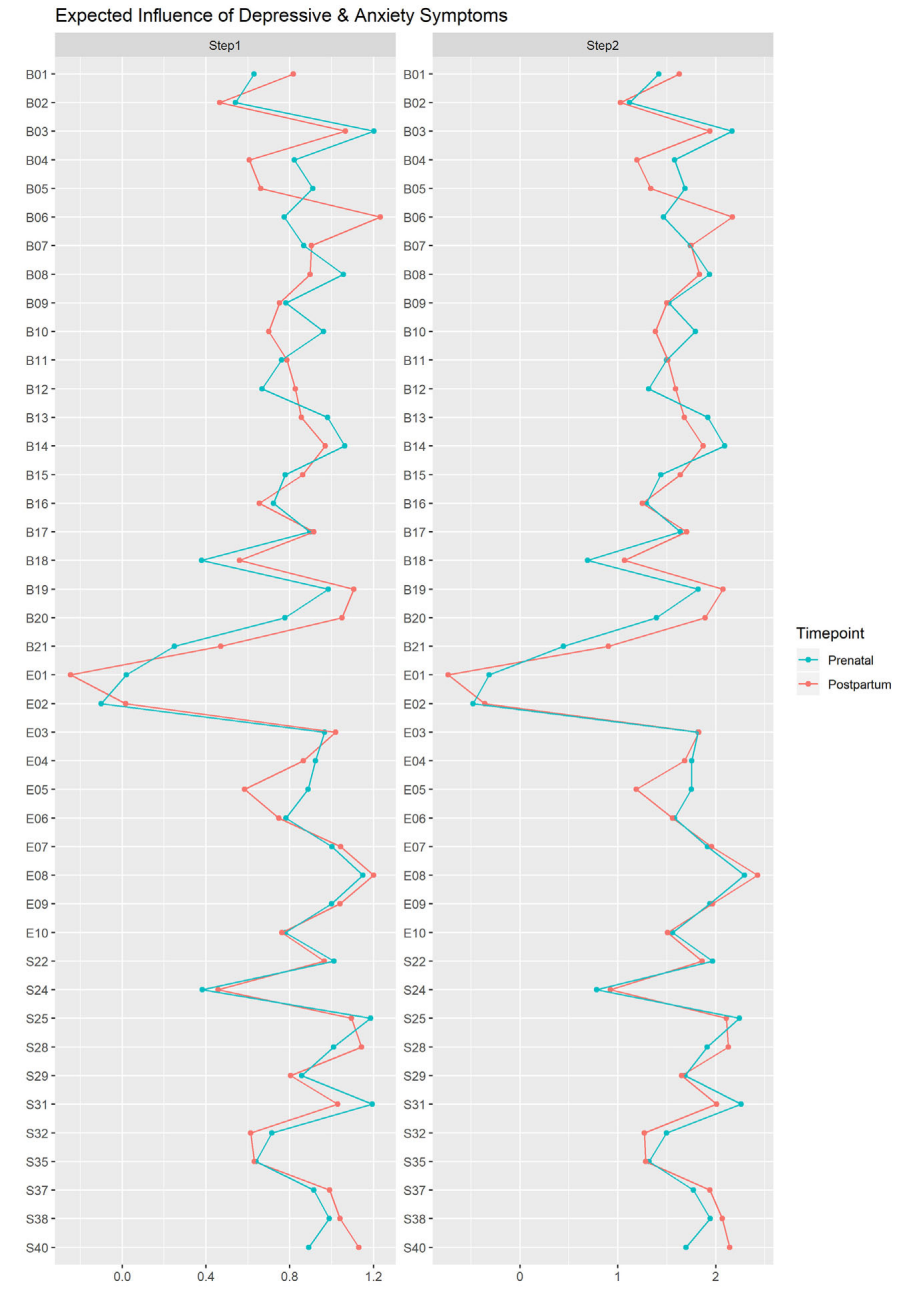
Z scores of the one-step and two-step expected indices of depressive and anxiety symptoms in the prenatal and post-partum networks. See **Figure 1** for the wordings of the symptoms.

The one-step EI indices of the prenatal (CS-EI1 = 0.35) and post-partum (CS-EI1 = 0.40) depressive-anxiety networks exceeded the recommended threshold and were thus stable. The prenatal and post-partum symptom one-step (*r* = 0.85, *p* <.001) and two-step (*r* = 0.90, *p* <.001) EI indices (**Figure 3**) were significantly correlated, suggesting an overall temporal stability of the symptom-symptom connectivity within the depressive-anxiety networks.

However, there were notable changes in the EI indices of specific symptoms. The permutation hypothesis test revealed seven symptoms that showed significant changes (*p* <.05) in EI1 (**Table 2**). In addition, of the top five prenatal and post-partum symptoms with the highest EI1 indices, four of them were different (**Table S3** in Supplementary Material). During pregnancy, the top five symptoms were “B03: Past Failure,” “B14: Worthlessness,” “E08: I have felt sad or miserable,” “S25: I feel like a failure,” and “S31: I have disturbing thoughts.” After pregnancy, the five symptoms with highest EI1 values were “B06: Punishment feelings,” “B19: Concentration difficulty,” “E08: I have felt sad or miserable,” “S28: I feel that difficulties are piling up so that I cannot overcome them,” and “S40: I get in a state of tension/turmoil as I think over my recent concerns and interests.” These changes in central symptoms suggested a change in the depressive-anxiety profiles before and after delivery.

**Table 2:** One-step EI *z*-scores and *p* values of depressive-anxiety symptoms that were significantly different (*p* <.05) between the prenatal and post-partum timepoints.

Symptom	Expected influence (*z* score)		*p*
	Prenatal	Post-partum	
B06: Punishment feelings	0.77	1.23	.002
B18: Changes in appetite	0.38	0.56	.026
B19: Concentration difficulty	0.98	1.10	.026
B20: Tiredness or fatigue	0.78	1.05	.006
B21: Loss of interest in sex	0.25	0.47	.049
E05: I have felt scared or panicky for no very good reason.	0.89	0.59	.002
S40: I get in a state of tension/turmoil as I think over my recent concerns and interests.	0.89	1.13	.037

The p values were based on permutation hypothesis test of difference in raw one-step EI indices between prenatal and post-partum networks that were individually estimated.

#### Predictive Value of Central Symptoms

The predictive values of symptoms were derived from zero-order correlations between ratings of individual symptoms and the respective birth (**Table S4**) and child outcomes (**Table S5**). Higher absolute predictive values indicate a stronger association between the rating of the maternal depressive-anxiety symptom and offspring development. The predictive values were regressed on the corresponding prenatal symptom EI index (EI1) as a test for the predictive value of symptom centrality.

**Figure 4:**
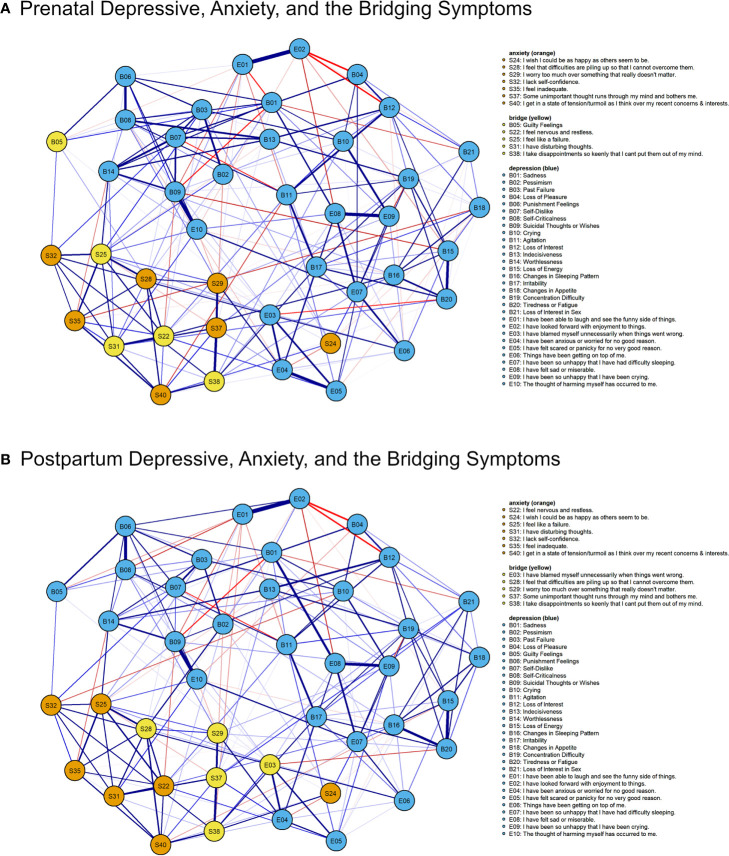
Symptoms that bridged maternal depression and anxiety in the prenatal **(A)** and post-partum **(B)** networks. Only the five symptoms with strongest bridge EI are shown.

EI1 of symptoms during pregnancy was significantly associated with the predictive value of gestational age (*β* = -0.43, *p* = .005; **Figure 4**) but not with the predictive values of birth weight (*β* = -0.03, *p* = .86) nor birth length (*β* = -0.30, *p* = .055). EI1 of the prenatal depressive-anxiety symptoms were significantly associated with the internalizing (*β* = 0.59, *p* <.001) and externalizing (*β* = 0.47, *p* = .002) symptoms of the child at 24 months old, composite IQ score at 54 months old (*β* = -0.38, *p* = .014), and spatial working memory errors at 72 months old (*β* = 0.52, *p* <.001). There was no significant association with stop-signal-task performance of the child at 72 months (*β* = 0.03, *p* = .84). In general, symptoms that were higher in EI during pregnancy were associated with poorer birth and child outcomes.

### Bridging Symptoms

#### Depressive-Anxiety Symptoms

Bridge EI indicates that the number and strength of direct (bEI1) and indirect (bEI2) connections a symptom have with symptoms from another community (e.g., depressive symptom with anxiety symptoms). There were significant correlations between the bridge EI indices of the prenatal and post-partum depressive-anxiety symptoms (bEI1: *r* = .74, *p* <.001; bEI2: *r* = .84, *p* <.001; **Figure S3**). We extracted the top five bridging symptoms (i.e., 90^th^ percentile) in the prenatal and post-partum depressive-anxiety networks (**Figure 4**). Four of the five symptoms were anxiety symptoms in both the prenatal and post-partum networks. Only the symptom “S38: I take disappointments so keenly that I can’t put them out of my mind” remained as the top five bridging symptoms during and after pregnancy. The strongest depressive bridging symptom was “B05: Guilty feelings” (0.28) and the strongest anxiety bridging symptom was “S25: I feel like a failure” (0.38). After pregnancy, the strongest depressive symptom was “E03: I have blamed myself unnecessarily when things went wrong” (0.31) and the strongest anxiety bridging symptom was “S28: I feel that difficulties are piling up so that I cannot overcome them” (0.36). Changes in strong bridging symptoms suggest that the symptom (e.g., depressive symptom) that affected or was affected by symptoms of another disorder (e.g., anxiety) was different across the peripartum period.

#### Negative and Positive Symptoms

We were also interested in the symptoms that bridged the negative (i.e., depression and anxiety) and positive dimensions of mental health. Bridge EI indices were calculated with negative mental health (i.e., depression and anxiety) symptoms as one community and the positive mental health symptoms as the other community. The prenatal and post-partum bridge EI indices of the negative and positive mental health symptoms were significantly correlated (bEI1: *r* = .90, *p* <.001; bEI2: *r* = .93, *p* <.001; **Figure S4**). During pregnancy, the strongest bridging symptoms for negative and positive mental health were “E08: I have felt sad or miserable” (bEI1 = -0.35) and “S34: I make decisions easily” (bEI1 = -0.70), respectively. After pregnancy, the strongest negative and positive mental health bridging symptoms were “B04: Loss of Pleasure” (bEI1 = -0.46) and “S27: I am calm, cool, and collected” (bEI1 = -0.64), respectively. The associations between negative and positive mental health changed across the peripartum period.

## Discussion

This study examined the networks of prenatal and post-partum depressive-anxiety symptoms. The symptom network analysis provided a deeper understanding of the dynamic changes in maternal symptoms of depression and anxiety occurring across the pre- and post-partum periods. Despite the profound differences in maternal circumstances and demands across the peripartum period, this analysis is, to our knowledge, the first to describe variation in symptom profiles. Our analysis demonstrates the use of symptom network analysis to conceptualize maternal mental health as an emergent construct of interrelated and mutually reinforcing symptoms. The results demonstrate that the circumstance of motherhood associated with changes in the presentation of maternal mental health symptoms.

### Network Structure

While there was no change in the structure of the depressive-anxiety symptoms during and after pregnancy, the strength of the connections between the symptoms was stronger post-partum. The increased connectivity may reflect the mutual reinforcement of depression and anxiety symptoms over the course of pregnancy and motherhood. Connections among the symptoms will become stronger as they keep reinforcing each other over time (77, 78). Anxiety symptoms during pregnancy predicted depressive symptoms post-partum; prenatal depressive symptoms likewise predicted post-partum anxiety symptoms (79). Similar results of decreasing depressive symptom score with increased connectivity between symptoms have been demonstrated in a general psychiatric sample (28). Clinically, this finding supports the notion of treating depressive-anxiety symptoms prenatally, as the denser post-partum network suggests poorer prognosis after pregnancy. Stronger feedback among the post-partum symptoms may result in greater vulnerability to clinical levels of depression or anxiety with poorer recovery prospects (80).

### Symptoms Centrality

There were qualitative differences in core depressive-anxiety symptoms across the peripartum periods as demonstrated in the change of the top central symptoms. This finding adds to the evidence from other studies reporting differences in pre- and post-partum depression profiles with the structured clinical interview (81, 82). During pregnancy, the cognitive-affective symptoms were more central. These symptoms were related to feeling worthless, agonizing over past failures, or having disturbing thoughts. This finding is consistent with reports describing that affective symptoms were more indicative of prenatal depression than were other symptoms (83). After pregnancy, the sense of being overwhelmed or being punished were most central in the depressive-anxiety network. The struggles of meeting the heightened demands of being a mother, together with a sense of incompetence or feelings of being punished for being a bad mother, are core features of post-partum depression (84). These findings suggest that negative self-perceptions (e.g., low self-esteem and self-blame) may be targets for clinical consideration during pregnancy, but skill-based interventions that help mothers cope with the demands of parenthood may be more important post-partum.

#### Predictive Value

We also tested the predictive value of centrality on birth and child outcomes. Central depressive-anxiety symptoms (i.e., higher EI values) during pregnancy were associated with worse birth and development outcomes for the child, including lower gestational age, higher internalizing and externalizing problems, lower IQ score, and poorer spatial working memory. The detrimental effect of poor prenatal mental health on birth and child outcomes is well documented. The current study shows that not all mental health symptoms are equal in their impact on the offspring. As central symptoms, the sense of failure or worthlessness may be targets for more effective prenatal interventions. However, as this is an associative study, randomized controlled trials targeting specific symptom features will be necessary for more definitive conclusions on whether intervening on prenatal mental health symptoms that are high in centrality is more effective than interventions on less central symptoms.

### Bridging Symptoms

Bridging symptoms are symptoms strongly associated with other symptoms of the other “condition”. In the depressive-anxiety networks, most of the top bridging symptoms were anxiety symptoms. This corroborates with increasing findings of anxiety disorders preceding the development of comorbid depressive conditions (42). A meta-analysis similarly showed that anxiety disorders were slightly stronger predictors for depression than vice versa (85). Despite the impact of anxiety on developing comorbid depression, maternal anxiety has not been emphasized as heavily as depression in both literature and clinical practice (86, 87).

The top bridging symptoms were different during and after pregnancy. During pregnancy, the top bridging symptoms were related to feelings of guilt, nervousness, or being a failure. After pregnancy, the top bridging symptoms were related to self-blame, feeling overwhelmed, and excessive worries. This finding is consistent with the idea that the core features of post-partum depression derive from the difficulty in meeting heightened demands of motherhood, sense of incompetence, and inadequacy (84). Since comorbid depression and anxiety have the worst prognosis and impact on offspring development (42, 44), there is clearly a need to prevent maternal anxiety from feeding into depressive symptoms. However, the clinically relevant maternal anxiety symptoms differ during and after pregnancy due to the change in circumstances.

The top bridging symptoms between negative and positive mental health also change during and after pregnancy. During pregnancy, the top positive bridging symptom was being able to make decisions easily. After pregnancy, the top positive bridging symptom was being able to remain calm and collected, consistent with the need to meet increased demands of motherhood. Bassi et al. (48) also found slightly different positive mental health symptoms associated with prenatal and post-partum depression. During pregnancy, in preparation for the arrival of the child, there is a demand for planning and decisions. However, after pregnancy, taking care of a newborn results in a sudden increase in demands (88). Positive mental health is a strong protective factor against the development of mental health problems [e.g., (89–91)]. The identification of positive bridging symptoms allows us to take a proactive approach, through public health promotion efforts, in preventing maternal mental health issues arising from pregnancy or childrearing stresses.

### Limitations and Future Research

The network analyses conducted in this study were correlational and exploratory. As such, we cannot ascertain the directionality of the symptom-symptom interaction. A highly influential or central symptom may be a target for intervention because it affects many symptoms. However, a symptom may be statistically central as a result of being a common consequence of other symptoms. Future research on maternal mental health networks will benefit from directed networks estimated from longitudinal data of at least three time points. Moreover, this study is a between-subject design. Within-subject design with intensive sampling is required to determine if the results can be generalized to individuals. This exploratory analysis provides plausible hypotheses for future studies.

The current sample was limited to complete cases, as the network comparison tests did not allow for missing values. The use of complete cases may have introduced biases to the analyses [e.g., (92, 93)]. Moreover, there may be sampling bias due to attrition as mothers with higher depressive-anxiety symptoms are more likely to drop out of research studies (56). Replication and the use of multiple imputations in network analyses are necessary for more definitive conclusions on the symptom profiles of pre- and postnatal maternal mental health.

Our research used self-reported depression and anxiety scales. Future research should include others-reported or clinician-reported questionnaires to provide a more comprehensive understanding of maternal mental health. Self-reported (e.g., BDI) and clinician-reported (e.g., structured clinical interview for DSM-IV) questionnaires often have different items, thus covering different aspects of depression or anxiety (94). Moreover, both individual and clinician provide different clinically significant information regarding the person’s mental health (95, 96).

Our research does not inform on etiology. However, it does provide a framework for such analyses. Future research should include relevant environmental (e.g., traumatic life events), biological (e.g., polygenic risk score), cognitive (e.g., sensitivity to negative information), or cultural (e.g., cultural attitudes toward parenting) etiologies in the maternal mental health networks. Symptomology networks allow for a deeper analysis into the mechanisms that affect maternal mental health. For example, experiencing a death in the family may trigger specific depressive symptoms, such as insomnia or excessive crying, which activate other depressive symptoms. Such external events may also affect maternal mental health by lowering the threshold with which particular triggers activate mental health symptoms (97).

## Conclusion

This study expands the peripartum mental health literature by examining maternal depression and anxiety at the symptoms level. A symptom network perspective allows a more detailed description of the nuances of maternal mental health at different stages of motherhood reflected in unique symptom-symptom interactions. The differences in symptom-symptom interactions, central, and bridging symptoms between pre- and post-partum suggest that maternal mental health during and after pregnancy may have different presentations and etiologies. This deep phenotyping approach potentially forms the basis for future research on biological, cultural, and experiential (e.g., socioeconomic status and past trauma) mechanisms underlying prenatal and post-partum mental health and may be useful for targeted interventions. This study suggested the importance of managing prenatal anxiety symptoms to prevent the development of comorbid disorders. This study also suggested that skill-based interventions may be more relevant post-partum than prenatal.

## Data Availability Statement

The raw data supporting the conclusions of this article will be made available by the authors, without undue reservation.

## Ethics Statement

The studies involving human participants were reviewed and approved by Domain-Specific Review Board of NUH and the Centralized Institutional Review Board of KKH. Written informed consent to participate in this study was provided by the participants for themselves and their child.

## Author Contributions

DP did the statistical analysis and wrote the first draft of the manuscript. HC, YC, PG, BB, and MM contributed to the design of the study, its measures, and protocols. All authors contributed to the article and approved the submitted version.

## Funding

This research was supported by a Translational and Clinical Research (TCR) grant from the Singapore National Medical Research Council (NMRC), Singapore (NMRC/TCR/004-NUS/2008; NMRC/TCR/012-NUHS/2014). Additional funding was provided by the Singapore Institute for Clinical Sciences, Agency for Science Technology and Research (A*STAR), Singapore. MM is supported by the Hope for Depression Research Foundation and the Jacobs Foundation.

## Conflict of Interest

The authors declare that the research was conducted in the absence of any commercial or financial relationships that could be construed as a potential conflict of interest.

## References

[1] Meaney MJ Perinatal maternal depressive symptoms as an issue for population health. Am J Psychiatry (2018) 175(11):1084–93. 10.1176/appi.ajp.2018.17091031 30068258

[2] Goodman SH Rouse MH Connell AM Broth MR Hall CM Heyward D Maternal depression and child psychopathology: A meta-analytic review. Clin Child Fam Psychol Rev (2011) 14(1):1–27. 10.1007/s10567-010-0080-1 21052833

[3] Pearson RM Bornstein MH Cordero M Scerif G Mahedy L Evans J Maternal perinatal mental health and offspring academic achievement at age 16: the mediating role of childhood executive function. J Child Psychol Psychiatry (2016) 57(4):491–501. 10.1111/jcpp.12483 26616637 PMC4789117

[4] Netsi E Pearson RM Murray L Cooper P Craske MG Stein A Association of persistent and severe postnatal depression with child outcomes. JAMA Psychiatry (2018) 75(3):247–53. 10.1001/jamapsychiatry.2017.4363 PMC5885957 29387878

[5] World Health Organization Millennium Development Goal 5: Improving maternal mental health. Geneva, Switzerland: Abuse DoMHaS (2008).

[6] Glover V Maternal depression, anxiety and stress during pregnancy and child outcome; what needs to be done. Best Pract Res Clin Obstetrics Gynaecol (2014) 28(1):25–35. 10.1016/j.bpobgyn.2013.08.017 24090740

[7] O’Donnell KJ Meaney MJ Fetal origins of mental health: The developmental origins of health and disease hypothesis. Am J Psychiatry (2017) 174(4):319–28. 10.1176/appi.ajp.2016.16020138 27838934

[8] Bauer A Parsonage M Knapp M Lemmi V Adelaja B The costs of perinatal mental health problems. (2014). Available from: http://eprints.lse.ac.uk/59885/.

[9] Evans J Heron J Francomb H Oke S Golding J Cohort study of depressed mood during pregnancy and after childbirth. BMJ (2001) 323(7307):257–60. 10.1136/bmj.323.7307.257 PMC35345 11485953

[10] Heron J O’Connor TG Evans J Golding J Glover V The course of anxiety and depression through pregnancy and the postpartum in a community sample. J Affect Disord (2004) 80(1):65–73. 10.1016/j.jad.2003.08.004 15094259

[11] Cents RAM Diamantopoulou S Hudziak JJ Jaddoe VWV Hofman A Verhulst FC Trajectories of maternal depressive symptoms predict child problem behaviour: The Generation R Study. Psychol Med (2013) 43(1):13–25. 10.1017/S0033291712000657 22490169

[12] Santos H Tan X Salomon R Heterogeneity in perinatal depression: how far have we come? A systematic review. Arch Women’s Ment Health (2017) 20:11–3. 10.1007/s00737-016-0691-8 PMC5507213 27796597

[13] Mughal MK Giallo R Arnold P Benzies K Kehler H Bright K Trajectories of maternal stress and anxiety from pregnancy to three years and child development at 3 years of age: Findings from the All Our Families (AOF) pregnancy cohort. J Affect Disord (2018) 234:318–26. 10.1016/j.jad.2018.02.095 29604550

[14] Campbell SB Cohn JF Flanagan C Popper S Meyers T Course and correlates of postpartum depression during the transition to parenthood. Dev Psychopathol (1992) 4(1):29–47. 10.1017/S095457940000554X

[15] Figueiredo B Conde A Anxiety and depression symptoms in women and men from early pregnancy to 3-months postpartum: Parity differences and effects. J Affect Disord (2011) 132(1):146–57. 10.1016/j.jad.2011.02.007 21420178

[16] Coyne JC Toward an interactional description of depression. Psychiatry (1976) 39(1):28–40. 10.1080/00332747.1976.11023874 1257353

[17] Ross LE Sellers EM Gilbert Evans SE Romach MK Mood changes during pregnancy and the postpartum period: Development of a biopsychosocial model. Acta Psychiatr Scand (2004) 109(6):457–66. 10.1111/j.1600-0047.2004.00296.x 15117291

[18] Gotlib IH Whiffen VE Mount JH Milne K Cordy NI Prevalence rates and demographic characteristics associated with depression in pregnancy and the postpartum. J Consult Clin Psychol (1989) 57(2):269–74. 10.1037/0022-006X.57.2.269 2785127

[19] Martini J Petzoldt J Einsle F Beesdo-Baum K Höfler M Wittchen H-U Risk factors and course patterns of anxiety and depressive disorders during pregnancy and after delivery: A prospective-longitudinal study. J Affect Disord (2015) 175:385–95. 10.1016/j.jad.2015.01.012 25678171

[20] Mullarkey MC Stein AT Pearson R Beevers CG Network analyses reveal which symptoms improve (or not) following an Internet intervention (Deprexis) for depression. Depress Anxiety (2020) 37(2):115–24. 10.1002/da.22972 PMC6992506 31710772

[21] Borsboom D A network theory of mental disorders. World Psychiatry (2017) 16(1):5–13. 10.1002/wps.20375 28127906 PMC5269502

[22] Fried EI van Borkulo CD Cramer AO Boschloo L Schoevers RA Borsboom D Mental disorders as networks of problems: a review of recent insights. Soc Psychiatry Psychiatr Epidemiol (2017) 52(1):1–10. 10.1007/s00127-016-1319-z 27921134 PMC5226976

[23] Kendler KS Zachar P Craver C What kinds of things are psychiatric disorders? Psychol Med (2011) 41(6):1143–50. 10.1017/S0033291710001844 20860872

[24] Nuijten MB Deserno MK Cramer AOJ Borsboom D Mental disorders as complex networks. Clin Neuropsychiatry (2016) 13(4/5):68–76.

[25] Robinaugh DJ Hoekstra RHA Toner ER Borsboom D The network approach to psychopathology: A review of the literature 2008–2018 and an agenda for future research. Psychol Med (2020) 50(3):353–66. 10.1017/S0033291719003404 PMC7334828 31875792

[26] Borsboom D Rhemtulla M Cramer AOJ van der Maas HLJ Scheffer M Dolan CV Kinds versus continua: A review of psychometric approaches to uncover the structure of psychiatric constructs. Psychol Med (2016) 46(8):1567–79. 10.1017/S0033291715001944 26997244

[27] Cramer AOJ Waldorp LJ van der Maas HLJ Borsboom D Comorbidity: A network perspective. Behav Brain Sci (2010) 33(2-3):137–50. 10.1017/S0140525X09991567 20584369

[28] Beard C Millner AJ Forgeard MJC Fried EI Hsu KJ Treadway MT Network analysis of depression and anxiety symptom relationships in a psychiatric sample. Psychol Med (2016) 46(16):3359–69. 10.1017/S0033291716002300 PMC5430082 27623748

[29] McElroy E Patalay P In search of disorders: Internalizing symptom networks in a large clinical sample. J Child Psychol Psychiatry (2019) 60(8):897–906. 10.1111/jcpp.13044 30900257 PMC6767473

[30] Keller MC Neale MC Kendler KS Association of Different Adverse Life Events With Distinct Patterns of Depressive Symptoms. Am J Psychiatry (2007) 164(10):1521–9. 10.1176/appi.ajp.2007.06091564 17898343

[31] Lux V Kendler KS Deconstructing major depression: A validation study of the DSM-IV symptomatic criteria. Psychol Med (2010) 40(10):1679–90. 10.1017/S0033291709992157 PMC3010198 20059797

[32] Fried EI Nesse RM Depression is not a consistent syndrome: An investigation of unique symptom patterns in the STAR*D study. J Affect Disord (2015) 172:96–102. 10.1016/j.jad.2014.10.010 25451401 PMC4397113

[33] Chekroud AM Gueorguieva R Krumholz HM Trivedi MH Krystal JH McCarthy G Reevaluating the efficacy and predictability of antidepressant treatments: a symptom clustering approach. JAMA Psychiatry (2017) 74(4):370–8. 10.1001/jamapsychiatry.2017.0025 PMC5863470 28241180

[34] Putnam K Robertson-Blackmore E Sharkey K Payne J Bergink V Munk-Olsen T Heterogeneity of postpartum depression: a latent class analysis. Lancet Psychiatry (2015) 2(1):59–67. 10.1016/S2215-0366(14)00055-8 26359613 PMC4800818

[35] Barker ED The duration and timing of maternal depression as a moderator of the relationship between dependent interpersonal stress, contextual risk and early child dysregulation. Psychol Med (2013) 43(8):1587–96. 10.1017/S0033291712002450 PMC4104613 23127350

[36] Campbell SB Matestic P von Stauffenberg C Mohan R Kirchner T Trajectories of maternal depressive symptoms, maternal sensitivity, and children’s functioning at school entry. DP (2007) 43(5):1202–15. 10.1037/0012-1649.43.5.1202 17723045

[37] Kuo SY Yang YL Kuo PC Tseng CM Tzeng YL Trajectories of depressive symptoms and fatigue among postpartum women. J Obstetric Gynecol Neonatal Nurs (2012) 41(2):216–26. 10.1111/j.1552-6909.2011.01331.x 22375929

[38] Rouquette A Pingault J-B Fried EI Orri M Falissard B Kossakowski JJ Emotional and behavioral symptom network structure in elementary school girls and association with anxiety disorders and depression in adolescence and early adulthood: A network analysis. JAMA Psychiatry (2018) 75(11):1173–81. 10.1001/jamapsychiatry.2018.2119 PMC6248096 30128480

[39] Gorman JM Comorbid depression and anxiety spectrum disorders. Depress Anxiety (1996) 4(4):160–8. 10.1002/(SICI)1520-6394(1996)4:4<160::AID-DA2>3.0.CO;2-J 9166648

[40] Kaufman J Charney D Comorbidity of mood and anxiety disorders. Depress Anxiety (2000) 12(S1):69–76. 10.1002/1520-6394(2000)12:1+<69::AID-DA9>3.0.CO;2-K 11098417

[41] Sartorius N Üstün TB Lecrubier Y Wittchen H-U Depression comorbid with anxiety: Results from the who study on psychological disorders in primary health care. Br J Psychiatry (1996) 168(S30):38–43. 10.1192/S0007125000298395 8864147

[42] Falah-Hassani K Shiri R Dennis CL The prevalence of antenatal and postnatal co-morbid anxiety and depression: a meta-analysis. Psychol Med (2017) 47(12):2041–53. 10.1017/S0033291717000617 28414017

[43] Jones PJ Ma R McNally RJ Bridge centrality: A network approach to understanding comorbidity. Multivariate Behav Res (2019) 1–15. 10.1080/00273171.2019.1614898 31179765

[44] Ibanez G Charles M-A Forhan A Magnin G Thiebaugeorges O Kaminski M Depression and anxiety in women during pregnancy and neonatal outcome: Data from the EDEN mother–child cohort. Early Hum Dev (2012) 88(8):643–9. 10.1016/j.earlhumdev.2012.01.014 22361259

[45] de Cates A Stranges S Blake A Weich S Mental well-being: An important outcome for mental health services? Br J Psychiatry (2015) 207(3):195–7. 10.1192/bjp.bp.114.158329 26329562

[46] Huppert FA Whittington JE Evidence for the independence of positive and negative well-being: Implications for quality of life assessment. Br J Health Psychol (2003) 8(1):107–22. 10.1348/135910703762879246 12643820

[47] Phua DY Kee MZL Meaney MJ Positive maternal mental health, parenting, and child development. Biol Psychiatry (2020) 87(4):328–37. 10.1016/j.biopsych.2019.09.028 31839213

[48] Bassi M Delle Fave A Cetin I Melchiorri E Pozzo M Vescovelli F Psychological well-being and depression from pregnancy to postpartum among primiparous and multiparous women. J Reprod Infant Psychol (2017) 35(2):183–95. 10.1080/02646838.2017.1290222 29517362

[49] Brandel M Melchiorri E Ruini C The dynamics of eudaimonic well-being in the transition to parenthood: Differences between fathers and mothers. J Family Issues (2018) 39(9):2572–89. 10.1177/0192513X18758344

[50] Fritz J Stochl J Fried EI Goodyer IM van Borkulo CD Wilkinson PO Unravelling the complex nature of resilience factors and their changes between early and later adolescence. BMC Med (2019) 17(1):203. 10.1186/s12916-019-1430-6 31722707 PMC6854636

[51] Soh SE Lee SSM Hoon SW Tan MY Goh A Lee BW The methodology of the GUSTO cohort study: A novel approach in studying pediatric allergy. Asia Pacific Allergy (2012) 2(2):144–8. 10.5415/apallergy.2012.2.2.144 PMC3345328 22701865

[52] Beck AT Ward CH Mendelson M Mock J Erbaugh J An inventory for measuring depression. Arch Gen Psychiatry (1961) 4(6):561–71. 10.1001/archpsyc.1961.01710120031004 13688369

[53] Cox JL Holden JM Sagovsky R Detection of postnatal depression. Development of the 10-item Edinburgh Postnatal Depression Scale. Br J Psychiatry (1987) 150(6):782–6. 10.1192/bjp.150.6.782 3651732

[54] Fried EI The 52 symptoms of major depression: Lack of content overlap among seven common depression scales. J Affect Disord (2017) 208:191–7. 10.1016/j.jad.2016.10.019 27792962

[55] Spielberger CD Gorsuch RL Lushene RE Manual for the state-trait anxiety inventory. Palo Alto, CA: Consulting Psychologists Press (1970).

[56] Gunning MD Denison FC Stockley CJ Ho SP Sandhu HK Reynolds RM Assessing maternal anxiety in pregnancy with the State-Trait Anxiety Inventory (STAI): Issues of validity, location and participation. J Reprod Infant Psychol (2010) 28(3):266–73. 10.1080/02646830903487300

[57] Hernández-Martínez C Val VA Murphy M Busquets PC Sans JC Relation between positive and negative maternal emotional states and obstetrical outcomes. Women Health (2011) 51(2):124–35. 10.1080/03630242.2010.550991 21476173

[58] Kvaal K Laake K Anxiety and well-being in older people after discharge from hospital. J Adv Nurs (2003) 44(3):271–7. 10.1046/j.1365-2648.2003.02802.x 14641397

[59] Kvaal K Laake K Engedal K Psychometric properties of the state part of the Spielberger State-Trait Anxiety Inventory (STAI) in geriatric patients. Int J Geriatr Psychiatry (2001) 16(10):980–6. 10.1002/gps.458 11607943

[60] Phua DY Kee MKZL Koh DXP Rifkin-Graboi A Daniels M Chen H Positive maternal mental health during pregnancy associated with specific forms of adaptive development in early childhood: Evidence from a longitudinal study. Dev Psychopathol (2017) 29(5):1573–87. 10.1017/S0954579417001249 29162171

[61] Achenbach TM Manual for the young adult self-report and young adult behavior checklist. Burlington, Vermont: University of Vermont, Department of Psychiatry (1997).

[62] Kaufman AS Kaufman NL Kaufman Assessment Battery for Children. 2nd ed. Bloomington, MN: Pearson, Inc (2004).

[63] Costantini G Epskamp S EstimateGroupNetwork: Perform the join graphical lasso and selects tuning parameters. [R package] (2017). Available from: https://CRAN.R-project.org/package=EstimateGroupNetwork

[64] Costantini G Richetin J Preti E Casini E Epskamp S Perugini M Stability and variability of personality networks. A tutorial on recent developments in network psychometrics. Pers Individ Dif (2019) 136:68–78. 10.1016/j.paid.2017.06.011

[65] Danaher P Wang P Witten DM The joint graphical lasso for inverse covariance estimation across multiple classes. J Roy Stat Soc Ser B (Stat Method) (2014) 76(2):373–97. 10.1111/rssb.12033 PMC4012833 24817823

[66] Fried EI Eidhof MB Palic S Costantini G Huisman-van Dijk HM Bockting CLH Replicability and generalizability of Posttraumatic Stress Disorder (PTSD) networks: A cross-cultural multisite study of PTSD symptoms in four trauma patient samples. Clin psychol Sci (2018) 6(3):335–51. 10.1177/2167702617745092 PMC5974702 29881651

[67] Richetin J Preti E Costantini G De Panfilis C The centrality of affective instability and identity in Borderline Personality Disorder: Evidence from network analysis. PloS One (2017) 12(10):e0186695. 10.1371/journal.pone.0186695 29040324 PMC5645155

[68] Epskamp S Cramer AO Waldorp LJ Schmittmann VD Borsboom D qgraph: Network visualizations of relationships in psychometric data. J Stat Softw (2012) 48(4):1–18. 10.18637/jss.v048.i04

[69] Epskamp S Borsboom D Fried EI Estimating psychological networks and their accuracy: A tutorial paper. Behav Res Methods (2017) 50(1):195–212. 10.3758/s13428-017-0862-1 PMC5809547 28342071

[70] van Borkulo C Claudia van Borkulo (2018). [cited Jun 1 2019] Available from: https://cvborkulo.com/2018/05/12/updated-version-of-nct/.

[71] Moran MD Arguments for rejecting the sequential bonferroni in ecological studies. Oikos (2003) 100(2):403–5. 10.1034/j.1600-0706.2003.12010.x

[72] Rhemtulla M Fried EI Aggen SH Tuerlinckx F Kendler KS Borsboom D Network analysis of substance abuse and dependence symptoms. Drug Alcohol Depend (2016) 161:230–7. 10.1016/j.drugalcdep.2016.02.005 PMC4861635 26898186

[73] Robinaugh DJ Millner AJ McNally RJ Identifying highly influential nodes in the complicated grief network. JAP (2016) 125(6):747–57. 10.1037/abn0000181 PMC5060093 27505622

[74] Heeren A Jones PJ McNally RJ Mapping network connectivity among symptoms of social anxiety and comorbid depression in people with social anxiety disorder. J Affect Disord (2018) 228:75–82. 10.1016/j.jad.2017.12.003 29232567

[75] Jones PJ networktools: Tools for identifying important nodes in networks. [R package] (2019). Availbale from: https://CRAN.R-project.org/package=networktools

[76] Elliott H Jones PJ Schmidt U Central symptoms predict posttreatment outcomes and clinical impairment in anorexia nervosa: A network analysis. Clin psychol Sci (2019) 8(1):139–54 10.1177/2167702619865958

[77] McElroy E Fearon P Belsky J Fonagy P Patalay P Networks of depression and anxiety symptoms across development. J Am Acad Child Adolesc Psychiatry (2018) 57(12):964–73. 10.1016/j.jaac.2018.05.027 PMC6290121 30522742

[78] Van Der Maas HL Dolan CV Grasman RP Wicherts JM Huizenga HM Raijmakers ME A dynamical model of general intelligence: The positive manifold of intelligence by mutualism. PsychologR (2006) 113(4):842–61. 10.1037/0033-295X.113.4.842 17014305

[79] Skouteris H Wertheim EH Rallis S Milgrom J Paxton SJ Depression and anxiety through pregnancy and the early postpartum: An examination of prospective relationships. J Affect Disord (2009) 113(3):303–8. 10.1016/j.jad.2008.06.002 18614240

[80] van Borkulo C Boschloo L Borsboom D Penninx BW Waldorp LJ Schoevers RA Association of symptom network structure with the course of depression. JAMA Psychiatry (2015) 72(12):1219–26. 10.1001/jamapsychiatry.2015.2079 26561400

[81] Amiel Castro RT Pinard Anderman C Glover V O’Connor TG Ehlert U Kammerer M Associated symptoms of depression: Patterns of change during pregnancy. Arch Women’s Ment Health (2017) 20(1):123–8. 10.1007/s00737-016-0685-6 PMC5237451 27878386

[82] Kammerer M Marks MN Pinard C Taylor A von Castelberg B Künzli H Symptoms associated with the DSM IV diagnosis of depression in pregnancy and post partum. Arch Women’s Ment Health (2009) 12(3):135–41. 10.1007/s00737-009-0062-9 19337702

[83] Huffman LC Lamour M Bryan YE Pederson FA Depressive symptomatology during pregnancy and the postpartum period: Is the beck depression inventory applicable? J Reprod Infant Psychol (1990) 8(2):87–97. 10.1080/02646839008403614

[84] Knudson-Martin C Silverstein R Suffering in silence: A qualitative meta-data-analysis of postpartum depression. J Marital Fam Ther (2009) 35(2):145–58. 10.1111/j.1752-0606.2009.00112.x 19302513

[85] Jacobson NC Newman MG Anxiety and depression as bidirectional risk factors for one another: A meta-analysis of longitudinal studies. PsyB (2017) 143(11):1155–200. 10.1037/bul0000111 28805400

[86] Matthey S Barnett B Howie P Kavanagh DJ Diagnosing postpartum depression in mothers and fathers: Whatever happened to anxiety? J Affect Disord (2003) 74(2):139–47. 10.1016/S0165-0327(02)00012-5 12706515

[87] Miller RL Pallant JF Negri LM Anxiety and stress in the postpartum: Is there more to postnatal distress than depression? BMC Psychiatry (2006) 6(1):12. 10.1186/1471-244X-6-12 16563155 PMC1450275

[88] Brenning K Soenens B Mabbe E Vansteenkiste M Ups and downs in the joy of motherhood: Maternal well-being as a function of psychological needs, personality, and infant temperament. J Happiness Stud (2019) 20(1):229–50. 10.1007/s10902-017-9936-0

[89] Fredrickson BL Tugade MM Waugh CE Larkin GR What good are positive emotions in crisis? A prospective study of resilience and emotions following the terrorist attacks on the United States on September 11th, 2001. JPSP (2003) 84(2):365. 10.1037/0022-3514.84.2.365 PMC2755263 12585810

[90] Keyes CL Dhingra SS Simoes EJ Change in level of positive mental health as a predictor of future risk of mental illness. Am J Public Health (2010) 100(12):2366–71. 10.2105/AJPH.2010.192245 PMC2978199 20966364

[91] Lamers SM Westerhof GJ Glas CA Bohlmeijer ET The bidirectional relation between positive mental health and psychopathology in a longitudinal representative panel study. J Positive Psychol (2015) 10(6):553–60. 10.1080/17439760.2015.1015156

[92] van der Heijden GJMG T Donders AR Stijnen T Moons KGM Imputation of missing values is superior to complete case analysis and the missing-indicator method in multivariable diagnostic research: A clinical example. J Clin Epidemiol (2006) 59(10):1102–9. 10.1016/j.jclinepi.2006.01.015 16980151

[93] Knol MJ Janssen KJM Donders ART Egberts ACG Heerdink ER Grobbee DE Unpredictable bias when using the missing indicator method or complete case analysis for missing confounder values: an empirical example. J Clin Epidemiol (2010) 63(7):728–36. 10.1016/j.jclinepi.2009.08.028 20346625

[94] Rush AJ Carmody TJ Ibrahim HM Trivedi MH Biggs MM Shores-Wilson K Comparison of self-report and clinician ratings on two inventories of depressive symptomatology. Psychiatr Serv (2006) 57(6):829–37. 10.1176/ps.2006.57.6.829 16754760

[95] Uher R Perlis RH Placentino A Dernovšek MZ Henigsberg N Mors O Self-report and clinician-rated measures of depression severity: Can one replace the other? Depress Anxiety (2012) 29(12):1043–9. 10.1002/da.21993 PMC3750710 22933451

[96] Cuijpers P Li J Hofmann SG Andersson G Self-reported versus clinician-rated symptoms of depression as outcome measures in psychotherapy research on depression: A meta-analysis. Clin Psychol Rev (2010) 30(6):768–78. 10.1016/j.cpr.2010.06.001 20619943

[97] Fried EI Cramer AOJ Moving forward: Challenges and directions for psychopathological network theory and methodology. Perspect Psychol Sci (2017) 12(6):999–1020. 10.1177/1745691617705892 28873325

