# Supplementary material for: Network Analyses of Maternal Pre- and Post-Partum Symptoms of Depression and Anxiety
Source: Front Psychiatry. 2020 Aug 6;11:785. doi: 10.3389/fpsyt.2020.00785 (PMC7424069; doi:10.3389/fpsyt.2020.00785)
Supplement: Supplementary file 1 [file DataSheet_1.docx]

# Supplementary Figures


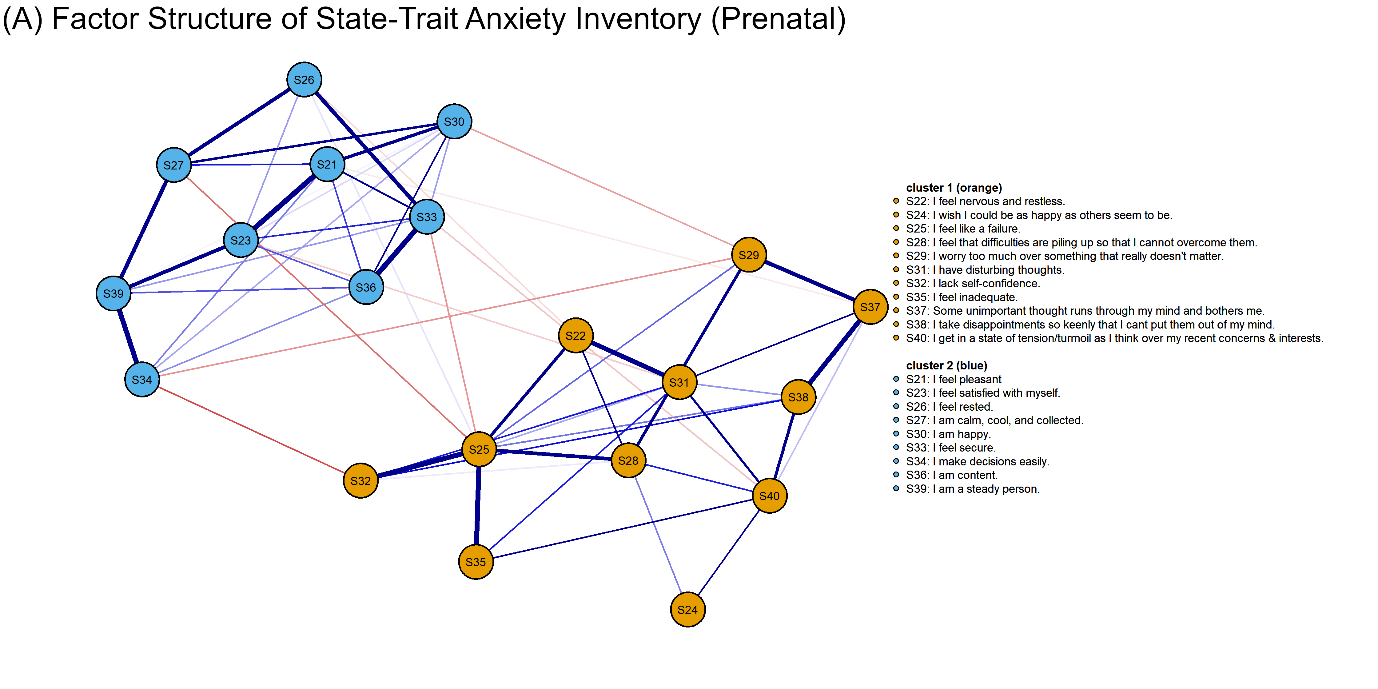


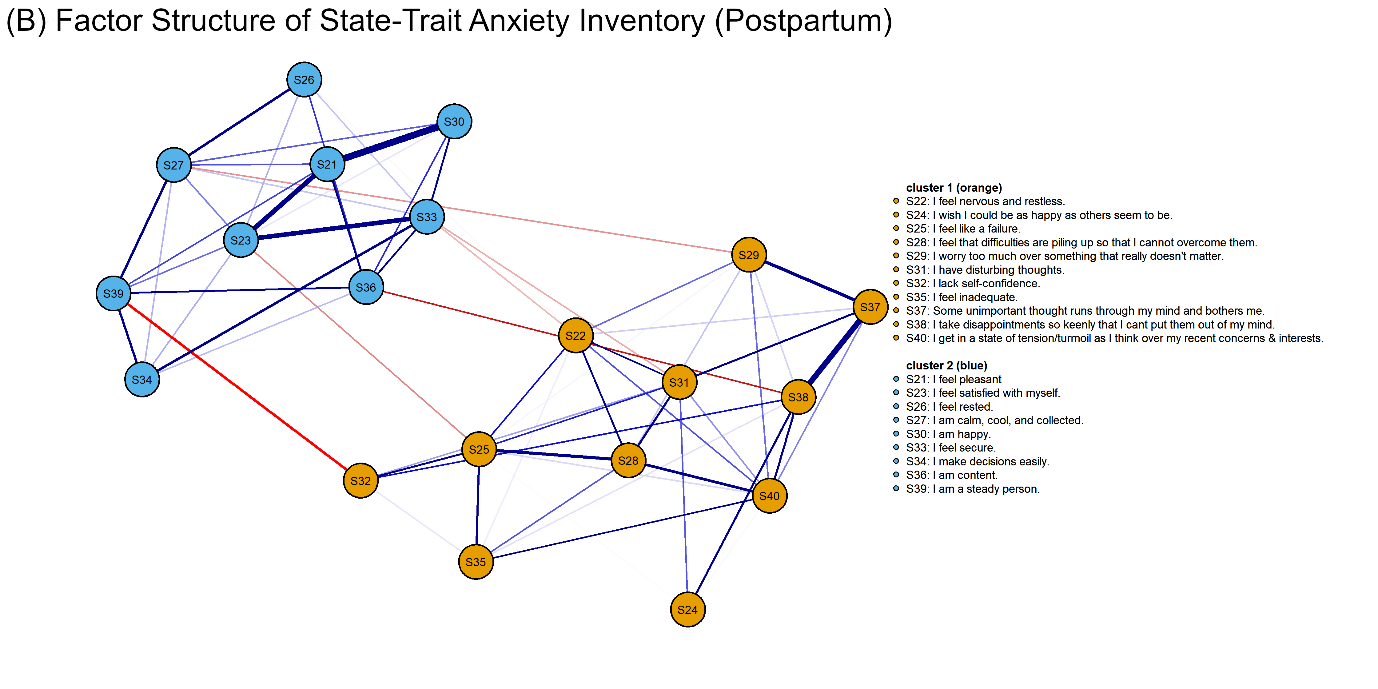


Figure S1. Exploratory factor structures of the prenatal (A) and postpartum (B) State-Trait Anxiety Inventory derived from exploratory graph analyses (Golino & Epskamp, 2017).


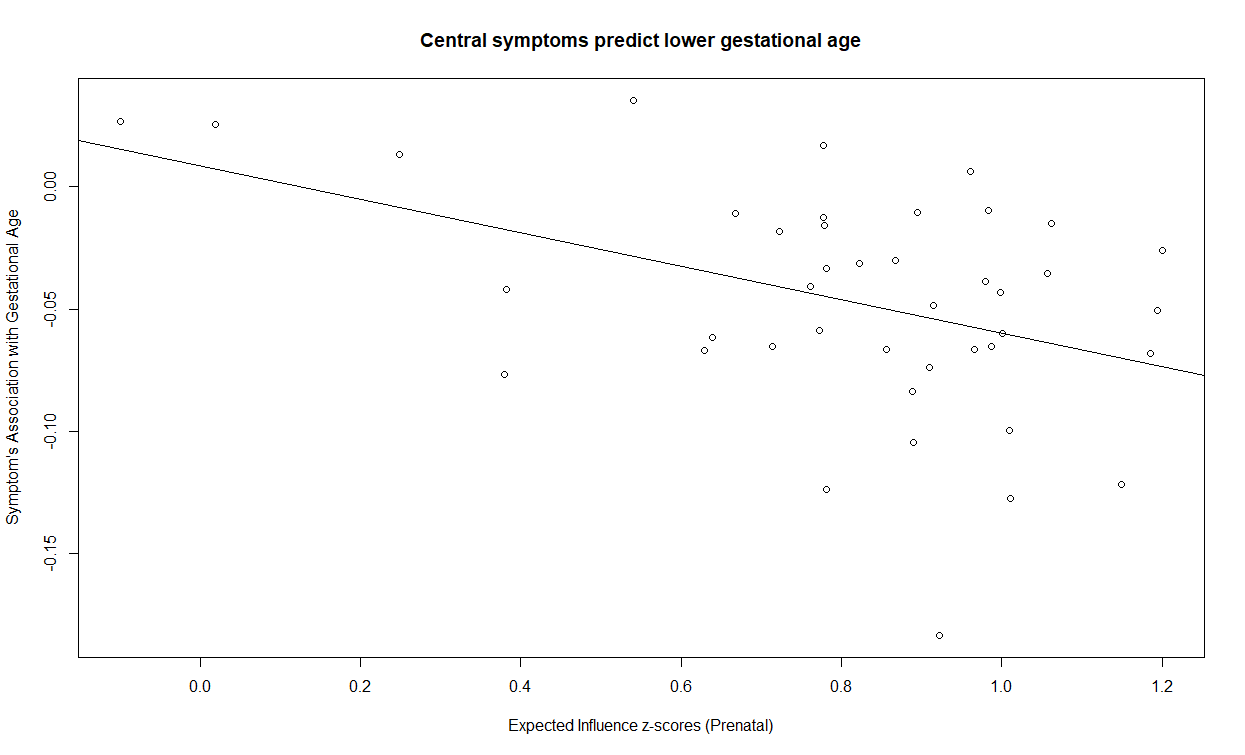


Figure S2. Effect of maternal depressive-anxiety symptom’s expected influence on its association on gestational age at birth. Each point represents a symptom during pregnancy. A point low on the *y*-axis represents a symptom that has high negative association with gestational age.


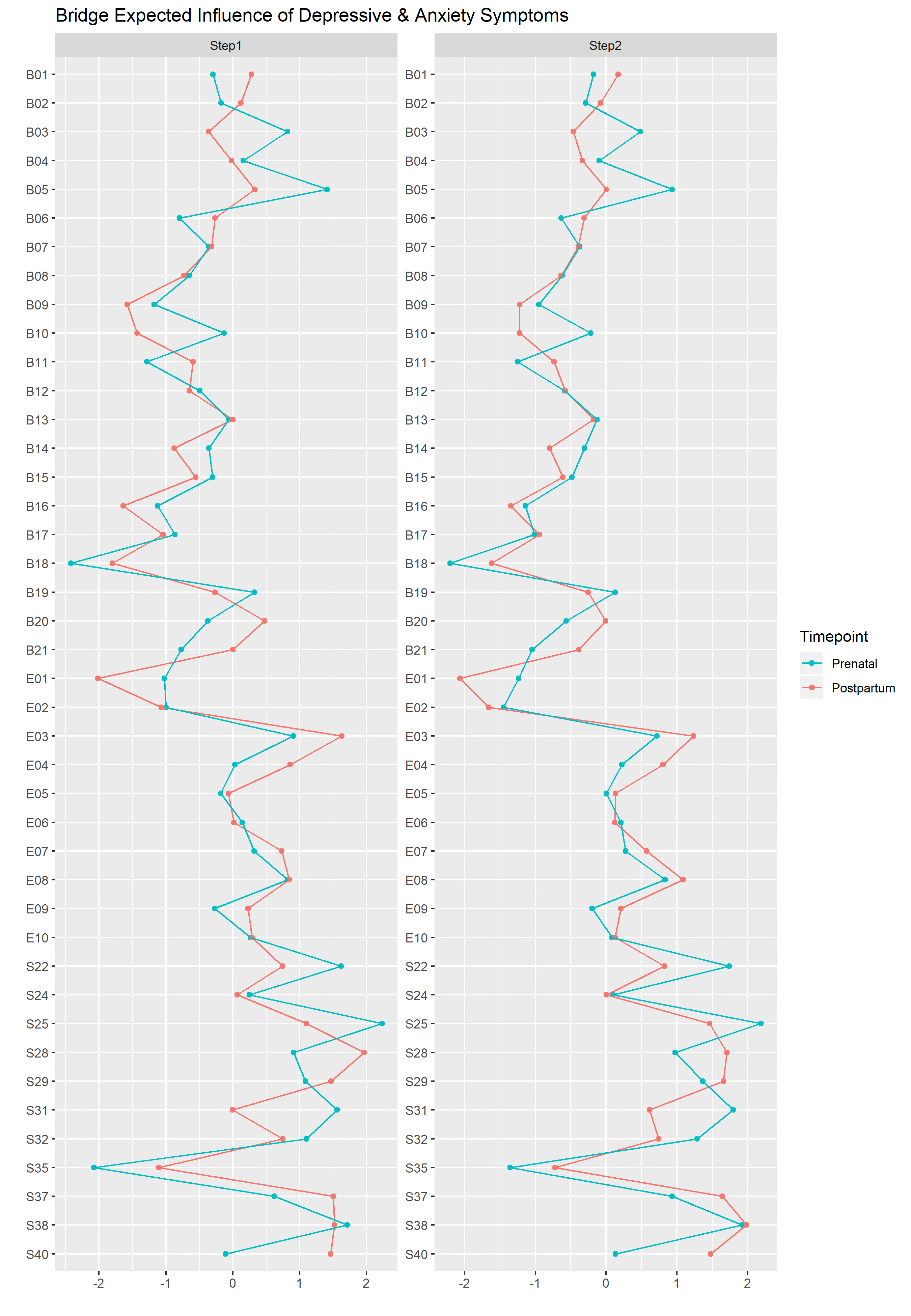


Figure S3. Standardized bridge expected influence of depressive-anxiety symptoms in prenatal and postpartum networks.


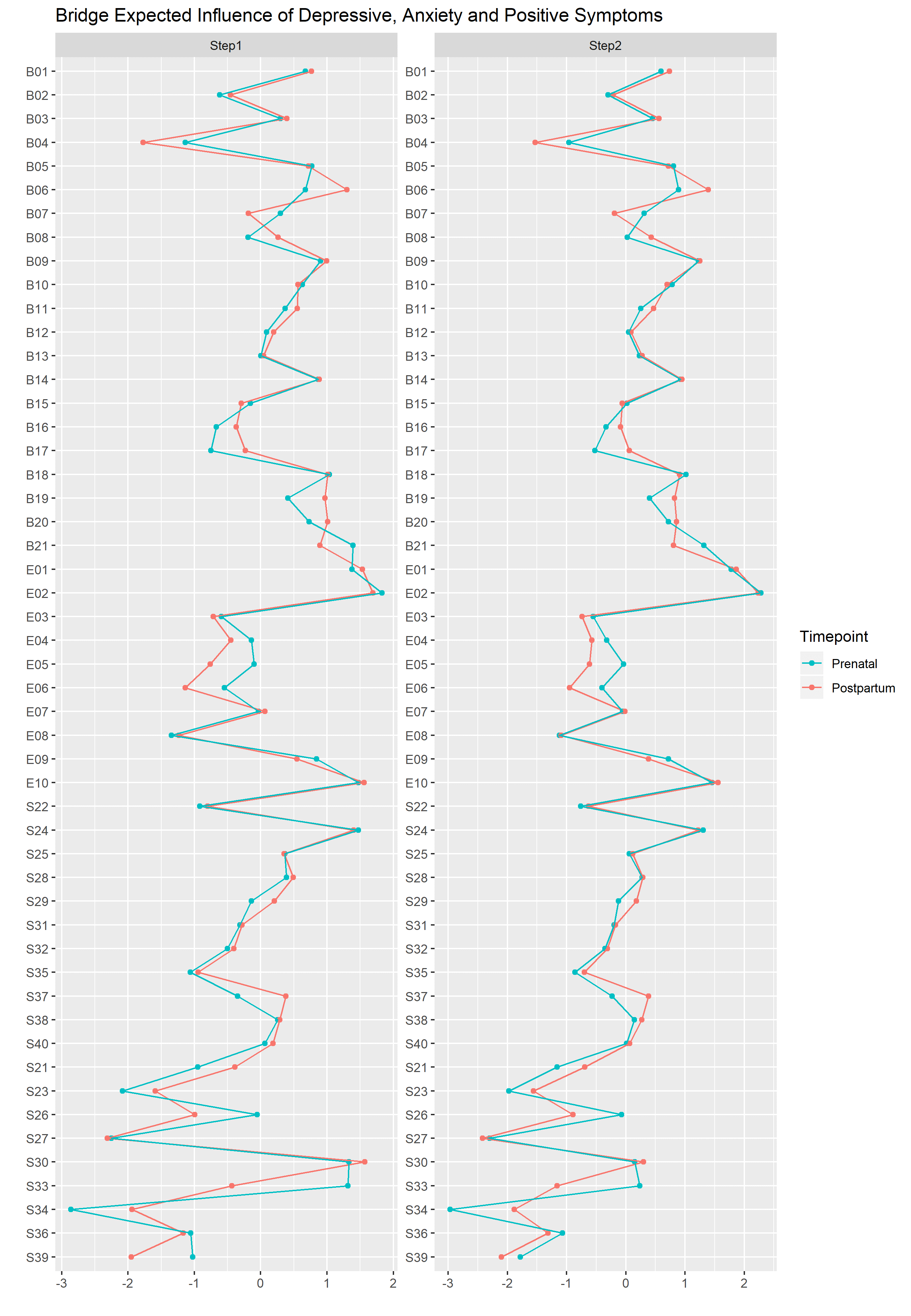


Figure S4. Standardized bridge expected influence of depressive-anxiety and positive mental health symptoms in prenatal and postpartum networks. Nodes S21 to S39 refers to the positive mental health items.

**Reference**

Golino, H. F., & Epskamp, S. (2017). Exploratory graph analysis: A new approach for estimating the number of dimensions in psychological research. *PloS One, 12*(6), e0174035. doi:10.1371/journal.pone.0174035
