# Supplementary material for: Network Analyses of Maternal Pre- and Post-Partum Symptoms of Depression and Anxiety
Source: Front Psychiatry. 2020 Aug 6;11:785. doi: 10.3389/fpsyt.2020.00785 (PMC7424069; doi:10.3389/fpsyt.2020.00785)
Supplement: Supplementary file 2 [file DataSheet_2.docx]

# Supplementary methods & materials

1. **Child outcome measures**

*1.1. Child Behavioral Checklist (CBCL)*

The CBCL is a common parent-rated screening tool for children’s behavioral and emotional problems (Achenbach, 1997). The different domains are classified into internalizing and externalizing symptoms. Internalizing symptoms included emotional reactivity, anxious/depressed, somatic complaints and being withdrawn. Externalizing symptoms included attention problems and aggressive behavior. Our variables of interest were the total raw scores of internalizing and externalizing problems.

*1.2. Kaufman Brief Intelligence Test (KBIT)*

The KBIT is an abbreviated intelligence measure for children (A. S. Kaufman & Kaufman, 2004). We used the composite IQ score as one of the measures of child development. The composite IQ is a standardized score that included the verbal and non-verbal components. The verbal component is a measure of crystallized intelligence and was assessed by Verbal Knowledge and Riddles sections of the KBIT. The non-verbal component is a measure of fluid reasoning and was assessed by the Matrics section.

*1.3. Spatial Working Memory (SWM) task*

The Spatial Working Memory task is part of the Cambridge Neuropsychological Test of Automated Battery (CANTAB; Cambridge Cognition). It was administered on a computer that had a touch-enabled screen attached to the monitor. During the task, several boxes were shown and the child was told to find blue tokens hidden within the boxes. To excel in this task, the child needed to maintain and update spatial information in the working memory during the search for the tokens (Luciana & Nelson, 2002). The variable of interest was the number of errors made when there were four to six boxes. An error was a revisit to a box that had already been searched. Higher number of errors suggests poorer spatial working memory.

*1.4. Stop-Signal Task (SST)*

The Stop-Signal task is a classic test of response inhibition consisting of Go and No-Go trials. During the Go trials, children saw an arrow on the screen and were instructed to indicate the direction of the arrow (i.e., the stimulus) by pressing either the left or right button on the press pad as quickly as possible. During the stop trials, children would hear a “beep” sound (i.e., the stop signal) and they needed to inhibit their response and not press any buttons. If inhibition was successful, the delay between the presentation of the stimulus and the stop signal (i.e., stop signal delay) increased. However, if the child failed to inhibit response, the stop signal delay decreased. The optimal stop signal delay was the length of delay with 50% success rate. The variable of interest was the Stop-Signal Reaction Time (SSRT) derived by subtracting the optimal stop signal delay from finishing time (Logan & Cowan, 1984). The SSRT indicates the time needed the child to initiate a stop response and a longer SSRT suggests worse inhibition.

**2. Statistical analysis**

We used the *goldbricker* function in the *networktools* R package to remove redundant items that measure the same construct (Jones, 2019). Overlapping items that measure the same construct will have similar patterns of correlations with other items. The inclusion of items with such topological similarity may result in inaccurate estimations of other correlations within the network (Fried & Cramer, 2017). The *goldbricker* function tests whether the correlations of a pair of items to another item are identical. We used the default settings of a threshold of 0.25 and 0.05 *p*-value cutoff with a minimum correlation coefficient of 0.50. Pairs of items will be considered as “bad” if 75% of the correlations to other items with other items are similar (at *p* < .05).

All depressive and anxiety items were retained for the network estimations as the *goldbricker* reported no statistically redundant items for both prenatal and postpartum measurements.

**References**

Achenbach, T. M. (1997). *Manual for the young adult self-report and young adult behavior checklist*: University of Vermont, Department of Psychiatry.

Kaufman, A. S., & Kaufman, N. L. (2004). *Kaufman Assessment Battery for Children* (2nd ed.). Bloomington, MN: Pearson, Inc.

Cambridge Cognition. CANTAB® [Cognitive assessment software]. All rights reserved. www.cantab.com.

Jones, P. J. (2019). networktools: Tools for identifying important nodes in networks. [R package]. Retrieved from https://CRAN.R-project.org/package=networktools

Luciana, M., & Nelson, C. A. (2002). Assessment of neuropsychological function through use of the Cambridge Neuropsychological Testing Automated Battery: Performance in 4- to 12-year-old children. *Developmental Neuropsychology, 22*(3), 595-624. doi:10.1207/S15326942DN2203_3

Logan, G. D., & Cowan, W. B. (1984). On the ability to inhibit thought and action: A theory of an act of control. *Psychological Review, 91*(3), 295-327. doi:10.1037/0033-295X.91.3.295
