# Supplementary material for: Network Analyses of Maternal Pre- and Post-Partum Symptoms of Depression and Anxiety
Source: Front Psychiatry. 2020 Aug 6;11:785. doi: 10.3389/fpsyt.2020.00785 (PMC7424069; doi:10.3389/fpsyt.2020.00785)
Supplement: Supplementary file 3 [file Table_1.docx]

# Supplementary Tables

**Table S1**. Sample demographics.

| **Variables** | ***n (%)*** | **Mean** | **Standard deviation** | **Range** |
| --- | --- | --- | --- | --- |
| Age |  | 30.72 | 4.8 | 19 – 43 |
| Age at which participants stopped formal education |  | 20.6 | 3.6 | 12 – 36 |
| First-time mother | 245 (49.9%) |  |  |  |
| Ethnicity |  |  |  |  |
| Chinese | 273 (55.6%) |  |  |  |
| Malays | 137 (27.9%) |  |  |  |
| Indians | 81 (16.5%) |  |  |  |

**Table S2.** Endorsement rates of the depressive, anxiety, and positive mental health symptoms.

| Items |  | Prenatal | | | |  | Postpartum | | | |
| --- | --- | --- | --- | --- | --- | --- | --- | --- | --- | --- |
| BDI |  | 0 | 1 | 2 | 3 |  | 0 | 1 | 2 | 3 |
| B01: Sadness |  | 0.82 | 0.17 | 0 | 0.01 |  | 0.83 | 0.15 | 0 | 0.01 |
| B02: Pessimism |  | 0.87 | 0.1 | 0.03 | 0 |  | 0.83 | 0.15 | 0.02 | 0.01 |
| B03: Past Failure |  | 0.86 | 0.07 | 0.06 | 0.01 |  | 0.87 | 0.08 | 0.04 | 0.01 |
| B04: Loss of Pleasure |  | 0.66 | 0.25 | 0.08 | 0.01 |  | 0.69 | 0.23 | 0.08 | 0.01 |
| B05: Guilty Feelings |  | 0.81 | 0.17 | 0.02 | 0 |  | 0.81 | 0.18 | 0 | 0 |
| B06: Punishment Feelings |  | 0.92 | 0.07 | 0 | 0.01 |  | 0.9 | 0.08 | 0.01 | 0.02 |
| B07: Self-Dislike |  | 0.92 | 0.07 | 0.01 | 0 |  | 0.87 | 0.09 | 0.04 | 0 |
| B08: Self-Criticalness |  | 0.87 | 0.09 | 0.02 | 0.02 |  | 0.82 | 0.13 | 0.02 | 0.02 |
| B09: Suicidal Thoughts or Wishes |  | 0.96 | 0.04 | 0 | 0 |  | 0.96 | 0.04 | 0 | 0 |
| B10: Crying |  | 0.75 | 0.16 | 0.07 | 0.03 |  | 0.81 | 0.12 | 0.02 | 0.04 |
| B11: Agitation |  | 0.65 | 0.3 | 0.03 | 0.02 |  | 0.7 | 0.26 | 0.01 | 0.02 |
| B12: Loss of Interest |  | 0.7 | 0.27 | 0.02 | 0.01 |  | 0.7 | 0.26 | 0.03 | 0.01 |
| B13: Indecisiveness |  | 0.79 | 0.18 | 0.02 | 0.01 |  | 0.79 | 0.19 | 0.02 | 0.01 |
| B14: Worthlessness |  | 0.92 | 0.06 | 0.01 | 0 |  | 0.93 | 0.05 | 0.02 | 0 |
| B15: Loss of Energy |  | 0.21 | 0.71 | 0.08 | 0 |  | 0.41 | 0.54 | 0.05 | 0 |
| B16: Changes in Sleeping Pattern |  | 0.21 | 0.59 | 0.16 | 0.03 |  | 0.26 | 0.56 | 0.16 | 0.01 |
| B17: Irritability |  | 0.59 | 0.35 | 0.05 | 0.01 |  | 0.67 | 0.3 | 0.03 | 0.01 |
| B18: Changes in Appetite |  | 0.26 | 0.58 | 0.14 | 0.01 |  | 0.51 | 0.41 | 0.06 | 0.02 |
| B19: Concentration Difficulty |  | 0.64 | 0.31 | 0.04 | 0.01 |  | 0.65 | 0.29 | 0.05 | 0 |
| B20: Tiredness or Fatigue |  | 0.12 | 0.78 | 0.08 | 0.02 |  | 0.37 | 0.57 | 0.05 | 0.01 |
| B21: Loss of Interest in Sex |  | 0.5 | 0.37 | 0.12 | 0.01 |  | 0.51 | 0.38 | 0.1 | 0.02 |
| EPDS |  | 0 | 1 | 2 | 3 |  | 0 | 1 | 2 | 3 |
| E01: I have been able to laugh and see the funny side of things. |  | 0 | 0.01 | 0.18 | 0.81 |  | 0 | 0.03 | 0.18 | 0.79 |
| E02: I have looked forward with enjoyment to things. |  | 0 | 0.02 | 0.21 | 0.77 |  | 0.01 | 0.02 | 0.21 | 0.77 |
| E03: I have blamed myself unnecessarily when things went wrong. |  | 0.22 | 0.49 | 0.25 | 0.04 |  | 0.27 | 0.45 | 0.24 | 0.04 |
| E04: I have been anxious or worried for no good reason. |  | 0.25 | 0.29 | 0.43 | 0.03 |  | 0.36 | 0.34 | 0.28 | 0.02 |
| E05: I have felt scared or panicky for no very good reason. |  | 0.38 | 0.42 | 0.18 | 0.02 |  | 0.49 | 0.31 | 0.17 | 0.03 |
| E06: Things have been getting on top of me. |  | 0.19 | 0.56 | 0.23 | 0.03 |  | 0.23 | 0.48 | 0.25 | 0.04 |
| E07: I have been so unhappy that I have had difficulty sleeping. |  | 0.46 | 0.33 | 0.18 | 0.03 |  | 0.6 | 0.25 | 0.13 | 0.01 |
| E08: I have felt sad or miserable. |  | 0.44 | 0.47 | 0.08 | 0.01 |  | 0.52 | 0.39 | 0.07 | 0.02 |
| E09: I have been so unhappy that I have been crying. |  | 0.55 | 0.38 | 0.06 | 0 |  | 0.58 | 0.37 | 0.04 | 0 |
| E10: The thought of harming myself has occurred to me. |  | 0.86 | 0.09 | 0.04 | 0.01 |  | 0.87 | 0.08 | 0.05 | 0 |
| STAI-negative |  | 1 | 2 | 3 | 4 |  | 1 | 2 | 3 | 4 |
| S22: I feel nervous and restless. |  | 0.41 | 0.53 | 0.05 | 0.01 |  | 0.5 | 0.45 | 0.04 | 0.02 |
| S24: I wish I could be as happy as others seem to be. |  | 0.3 | 0.35 | 0.22 | 0.13 |  | 0.33 | 0.35 | 0.19 | 0.14 |
| S25: I feel like a failure. |  | 0.8 | 0.16 | 0.03 | 0 |  | 0.73 | 0.23 | 0.03 | 0.01 |
| S28: I feel that difficulties are piling up so that I cannot overcome them. |  | 0.56 | 0.39 | 0.05 | 0.01 |  | 0.53 | 0.4 | 0.04 | 0.02 |
| S29: I worry too much over something that really doesn't matter. |  | 0.48 | 0.44 | 0.06 | 0.01 |  | 0.52 | 0.39 | 0.08 | 0.01 |
| S31: I have disturbing thoughts. |  | 0.51 | 0.42 | 0.07 | 0.01 |  | 0.55 | 0.38 | 0.04 | 0.02 |
| S32: I lack self-confidence. |  | 0.53 | 0.4 | 0.05 | 0.02 |  | 0.53 | 0.37 | 0.07 | 0.03 |
| S35: I feel inadequate. |  | 0.53 | 0.41 | 0.05 | 0.01 |  | 0.44 | 0.48 | 0.07 | 0.02 |
| S37: Some unimportant thought runs through my mind and bothers me. |  | 0.41 | 0.51 | 0.07 | 0.01 |  | 0.42 | 0.48 | 0.08 | 0.02 |
| S38: I take disappointments so keenly that I can’t put them out of my mind. |  | 0.49 | 0.41 | 0.08 | 0.01 |  | 0.51 | 0.39 | 0.07 | 0.03 |
| S40: I get in a state of tension/turmoil as I think over my recent concerns & interests. |  | 0.51 | 0.4 | 0.08 | 0.01 |  | 0.53 | 0.38 | 0.07 | 0.02 |
| STAI-positive |  | 1 | 2 | 3 | 4 |  | 1 | 2 | 3 | 4 |
| S21: I feel pleasant |  | 0 | 0.19 | 0.47 | 0.34 |  | 0.01 | 0.18 | 0.45 | 0.36 |
| S23: I feel satisfied with myself. |  | 0.02 | 0.19 | 0.48 | 0.3 |  | 0.02 | 0.2 | 0.44 | 0.34 |
| S26: I feel rested. |  | 0.05 | 0.41 | 0.41 | 0.13 |  | 0.08 | 0.41 | 0.35 | 0.16 |
| S27: I am calm, cool, and collected. |  | 0.02 | 0.3 | 0.46 | 0.22 |  | 0.05 | 0.29 | 0.41 | 0.25 |
| S30: I am happy. |  | 0 | 0.13 | 0.45 | 0.41 |  | 0 | 0.12 | 0.43 | 0.44 |
| S33: I feel secure. |  | 0.02 | 0.14 | 0.46 | 0.38 |  | 0.02 | 0.16 | 0.42 | 0.4 |
| S34: I make decisions easily. |  | 0.04 | 0.31 | 0.47 | 0.19 |  | 0.03 | 0.31 | 0.41 | 0.25 |
| S36: I am content. |  | 0.03 | 0.19 | 0.46 | 0.32 |  | 0.03 | 0.21 | 0.43 | 0.33 |
| S39: I am a steady person. |  | 0.02 | 0.21 | 0.53 | 0.25 |  | 0.02 | 0.23 | 0.44 | 0.31 |

*Note*. BDI = Beck Depression Inventory; EPDS = Edinburgh Postnatal Depression Scale; STAI = State-Trait Anxiety Inventory trait subscale. Negative refers to negatively worded items and is an indicator of anxiety level. Positive refers to positively worded items and is an indicator of positive mental health.

Table S3. One-step expected influence *z*-scores and *p*-values of depressive-anxiety symptoms.

| Symptom | Expected influence (*z­*-score) | | *p* |
| --- | --- | --- | --- |
|  | Prenatal | Postpartum |  |
| B01: Sadness | 0.63 | 0.82 | 0.78 |
| B02: Pessimism | 0.54 | 0.47 | 0.60 |
| B03: Past Failure | 1.20 | 1.07 | 0.11 |
| B04: Loss of Pleasure | 0.82 | 0.61 | 0.29 |
| B05: Guilty Feelings | 0.91 | 0.66 | 0.36 |
| **B06: Punishment Feelings** | **0.77** | **1.23** | **0.00** |
| B07: Self-Dislike | 0.87 | 0.90 | 0.81 |
| B08: Self-Criticalness | 1.06 | 0.90 | 0.69 |
| B09: Suicidal Thoughts or Wishes | 0.78 | 0.75 | 0.38 |
| **B10: Crying** | **0.96** | **0.70** | **0.15** |
| B11: Agitation | 0.76 | 0.79 | 0.83 |
| B12: Loss of Interest | 0.67 | 0.83 | 0.46 |
| B13: Indecisiveness | 0.98 | 0.85 | 0.64 |
| B14: Worthlessness | 1.06 | 0.97 | 0.65 |
| B15: Loss of Energy | 0.78 | 0.86 | 0.16 |
| B16: Changes in Sleeping Pattern | 0.72 | 0.66 | 0.88 |
| B17: Irritability | 0.90 | 0.92 | 0.95 |
| **B18: Changes in Appetite** | **0.38** | **0.56** | **0.03** |
| **B19: Concentration Difficulty** | **0.98** | **1.10** | **0.03** |
| **B20: Tiredness or Fatigue** | **0.78** | **1.05** | **0.01** |
| **B21: Loss of Interest in Sex** | **0.25** | **0.47** | **0.05** |
| E01: I have been able to laugh and see the funny side of things. | 0.02 | -0.25 | 0.55 |
| E02: I have looked forward with enjoyment to things. | -0.10 | 0.02 | 0.42 |
| E03: I have blamed myself unnecessarily when things went wrong. | 0.97 | 1.02 | 0.20 |
| E04: I have been anxious or worried for no good reason. | 0.92 | 0.86 | 0.84 |
| **E05: I have felt scared or panicky for no very good reason.** | **0.89** | **0.59** | **0.00** |
| E06: Things have been getting on top of me. | 0.78 | 0.75 | 0.31 |
| E07: I have been so unhappy that I have had difficulty sleeping. | 1.00 | 1.04 | 0.73 |
| E08: I have felt sad or miserable. | 1.15 | 1.20 | 0.46 |
| E09: I have been so unhappy that I have been crying. | 1.00 | 1.04 | 0.65 |
| E10: The thought of harming myself has occurred to me. | 0.78 | 0.76 | 0.41 |
| S22: I feel nervous and restless. | 1.01 | 0.96 | 0.89 |
| S24: I wish I could be as happy as others seem to be. | 0.38 | 0.46 | 0.26 |
| S25: I feel like a failure. | 1.19 | 1.09 | 0.14 |
| S28: I feel that difficulties are piling up so that I cannot overcome them. | 1.01 | 1.14 | 0.42 |
| S29: I worry too much over something that really doesn't matter. | 0.86 | 0.80 | 0.58 |
| S31: I have disturbing thoughts. | 1.19 | 1.03 | 0.11 |
| S32: I lack self-confidence. | 0.71 | 0.61 | 0.32 |
| S35: I feel inadequate. | 0.64 | 0.63 | 0.75 |
| S37: Some unimportant thought runs through my mind and bothers me. | 0.92 | 0.99 | 0.10 |
| S38: I take disappointments so keenly that I can’t put them out of my mind. | 0.99 | 1.04 | 0.93 |
| **S40: I get in a state of tension/turmoil as I think over my recent concerns & interests.** | **0.89** | **1.13** | **0.04** |

*Note*. The *p*-values were based on permutation hypothesis test of difference in raw one-step expected influence indices between prenatal and postpartum networks that were individually estimated. Bold indicates symptoms with statistically significant change in expected influence. Underline indicates the top five most influential prenatal and postpartum symptoms.

Table S4. Predictive values of maternal depressive-anxiety symptoms on birth outcomes.

| Symptom | Gestational age | Birth weight | Birth length |
| --- | --- | --- | --- |
| B01: Sadness | -0.067 | 0.034 | -0.027 |
| B02: Pessimism | 0.036 | 0.019 | -0.028 |
| B03: Past Failure | -0.026 | -0.023 | -0.063 |
| B04: Loss of Pleasure | -0.031 | 0.010 | -0.044 |
| B05: Guilty Feelings | -0.074 | -0.003 | -0.092 |
| B06: Punishment Feelings | -0.059 | -0.052 | -0.077 |
| B07: Self-Dislike | -0.030 | -0.035 | -0.049 |
| B08: Self-Criticalness | -0.036 | -0.045 | -0.107 |
| B09: Suicidal Thoughts or Wishes | -0.033 | -0.043 | -0.065 |
| B10: Crying | 0.007 | 0.068 | -0.032 |
| B11: Agitation | -0.041 | 0.064 | -0.022 |
| B12: Loss of Interest | -0.011 | 0.012 | 0.005 |
| B13: Indecisiveness | -0.038 | 0.003 | -0.056 |
| B14: Worthlessness | -0.015 | 0.018 | -0.029 |
| B15: Loss of Energy | -0.013 | 0.025 | 0.025 |
| B16: Changes in Sleeping Pattern | -0.018 | -0.037 | -0.046 |
| B17: Irritability | -0.010 | 0.049 | 0.024 |
| B18: Changes in Appetite | -0.077 | -0.063 | -0.062 |
| B19: Concentration Difficulty | -0.010 | 0.046 | -0.017 |
| B20: Tiredness or Fatigue | 0.017 | 0.016 | -0.027 |
| B21: Loss of Interest in Sex | 0.013 | 0.007 | -0.006 |
| E01: I have been able to laugh and see the funny side of things. | 0.026 | -0.014 | -0.008 |
| E02: I have looked forward with enjoyment to things. | 0.027 | -0.018 | -0.025 |
| E03: I have blamed myself unnecessarily when things went wrong. | -0.066 | -0.052 | -0.121 |
| E04: I have been anxious or worried for no good reason. | -0.184 | -0.076 | -0.217 |
| E05: I have felt scared or panicky for no very good reason. | -0.084 | -0.036 | -0.119 |
| E06: Things have been getting on top of me. | -0.124 | -0.042 | -0.115 |
| E07: I have been so unhappy that I have had difficulty sleeping. | -0.060 | -0.020 | -0.109 |
| E08: I have felt sad or miserable. | -0.122 | 0.024 | -0.045 |
| E09: I have been so unhappy that I have been crying. | -0.043 | 0.053 | -0.022 |
| E10: The thought of harming myself has occurred to me. | -0.016 | -0.007 | -0.020 |
| S22: I feel nervous and restless. | -0.127 | -0.020 | -0.124 |
| S24: I wish I could be as happy as others seem to be. | -0.042 | 0.051 | -0.024 |
| S25: I feel like a failure. | -0.068 | -0.009 | -0.009 |
| S28: I feel that difficulties are piling up so that I cannot overcome them. | -0.100 | -0.024 | -0.075 |
| S29: I worry too much over something that really doesn't matter. | -0.066 | 0.003 | -0.085 |
| S31: I have disturbing thoughts. | -0.051 | -0.011 | -0.060 |
| S32: I lack self-confidence. | -0.065 | -0.017 | -0.095 |
| S35: I feel inadequate. | -0.061 | -0.016 | -0.064 |
| S37: Some unimportant thought runs through my mind and bothers me. | -0.049 | -0.048 | -0.098 |
| S38: I take disappointments so keenly that I can’t put them out of my mind. | -0.065 | -0.072 | -0.065 |
| S40: I get in a state of tension/turmoil as I think over my recent concerns & interests. | -0.105 | -0.023 | -0.063 |

*Note.* Predictive values are zero-order spearman correlations between the symptom and birth outcome.

Table S5. Predictive values of maternal depressive-anxiety symptoms on child outcomes.

| Symptom | Internalizing symptoms | Externalizing symptoms | IQ | SST | SWM |
| --- | --- | --- | --- | --- | --- |
| B01: Sadness | 0.110 | 0.071 | -0.025 | -0.106 | 0.043 |
| B02: Pessimism | 0.153 | 0.249 | -0.112 | -0.016 | 0.012 |
| B03: Past Failure | 0.108 | 0.143 | -0.071 | 0.136 | 0.039 |
| B04: Loss of Pleasure | 0.218 | 0.283 | -0.084 | -0.077 | 0.113 |
| B05: Guilty Feelings | 0.128 | 0.121 | -0.051 | 0.016 | 0.088 |
| B06: Punishment Feelings | 0.062 | 0.046 | 0.108 | -0.019 | 0.212 |
| B07: Self-Dislike | 0.168 | 0.098 | -0.002 | 0.018 | -0.022 |
| B08: Self-Criticalness | 0.166 | 0.172 | -0.014 | -0.097 | 0.013 |
| B09: Suicidal Thoughts or Wishes | 0.129 | 0.095 | -0.082 | -0.010 | -0.077 |
| B10: Crying | 0.166 | 0.184 | 0.005 | -0.056 | 0.108 |
| B11: Agitation | 0.167 | 0.170 | 0.043 | -0.100 | 0.054 |
| B12: Loss of Interest | 0.216 | 0.303 | -0.020 | -0.142 | 0.145 |
| B13: Indecisiveness | 0.156 | 0.226 | -0.055 | 0.061 | 0.174 |
| B14: Worthlessness | 0.140 | 0.144 | -0.061 | 0.047 | 0.109 |
| B15: Loss of Energy | 0.276 | 0.295 | -0.006 | -0.167 | 0.055 |
| B16: Changes in Sleeping Pattern | 0.120 | 0.255 | 0.000 | -0.104 | 0.042 |
| B17: Irritability | 0.140 | 0.221 | 0.081 | -0.022 | 0.163 |
| B18: Changes in Appetite | 0.036 | 0.040 | -0.008 | 0.122 | -0.090 |
| B19: Concentration Difficulty | 0.177 | 0.211 | -0.016 | 0.085 | 0.216 |
| B20: Tiredness or Fatigue | 0.128 | 0.180 | -0.009 | -0.100 | 0.162 |
| B21: Loss of Interest in Sex | 0.101 | 0.103 | 0.000 | -0.136 | 0.028 |
| E01: I have been able to laugh and see the funny side of things. | -0.136 | -0.112 | 0.104 | -0.039 | -0.097 |
| E02: I have looked forward with enjoyment to things. | -0.132 | -0.156 | 0.048 | 0.086 | -0.085 |
| E03: I have blamed myself unnecessarily when things went wrong. | 0.076 | 0.094 | -0.073 | 0.114 | 0.049 |
| E04: I have been anxious or worried for no good reason. | 0.100 | 0.116 | 0.017 | -0.188 | 0.021 |
| E05: I have felt scared or panicky for no very good reason. | 0.237 | 0.172 | -0.040 | -0.097 | 0.031 |
| E06: Things have been getting on top of me. | 0.149 | 0.166 | -0.040 | 0.147 | 0.122 |
| E07: I have been so unhappy that I have had difficulty sleeping. | 0.154 | 0.080 | -0.201 | -0.041 | 0.055 |
| E08: I have felt sad or miserable. | 0.112 | 0.038 | -0.081 | -0.024 | 0.082 |
| E09: I have been so unhappy that I have been crying. | 0.114 | 0.078 | -0.077 | -0.085 | 0.106 |
| E10: The thought of harming myself has occurred to me. | 0.172 | 0.121 | -0.055 | -0.021 | 0.159 |
| S22: I feel nervous and restless. | 0.209 | 0.109 | -0.119 | -0.078 | 0.100 |
| S24: I wish I could be as happy as others seem to be. | 0.211 | 0.056 | -0.203 | 0.021 | -0.025 |
| S25: I feel like a failure. | 0.117 | 0.114 | -0.188 | 0.047 | 0.117 |
| S28: I feel that difficulties are piling up so that I cannot overcome them. | 0.184 | 0.095 | -0.065 | 0.013 | -0.008 |
| S29: I worry too much over something that really doesn't matter. | 0.159 | 0.166 | -0.004 | 0.046 | 0.207 |
| S31: I have disturbing thoughts. | 0.281 | 0.174 | -0.171 | -0.014 | 0.133 |
| S32: I lack self-confidence. | 0.231 | 0.207 | -0.034 | 0.077 | 0.159 |
| S35: I feel inadequate. | 0.124 | 0.070 | -0.146 | 0.051 | 0.073 |
| S37: Some unimportant thought runs through my mind and bothers me. | 0.160 | 0.170 | -0.064 | 0.030 | 0.162 |
| S38: I take disappointments so keenly that I can’t put them out of my mind. | 0.208 | 0.234 | -0.076 | -0.017 | 0.251 |
| S40: I get in a state of tension/turmoil as I think over my recent concerns & interests. | 0.176 | 0.110 | -0.157 | -0.004 | 0.153 |

*Note.* Externalizing and internalizing symptoms were mothers’ ratings on the Child Behavioral Checklist when the child was 24 months old. IQ score was assessed with the Kaufman Brief Intelligence Test at 54 months old. SST refers to Stop-Signal Task’s stop signal reaction time; higher reaction time indicates worse inhibition control. SWM refers to Spatial Working Memory task’s number of errors made during the 4-to-6 boxes stage; higher score indicates worse performance.
